# Effect of Glycosylation on the Enzymatic Degradation of D-Amino Acid-Containing Peptides

**DOI:** 10.3390/molecules30030441

**Published:** 2025-01-21

**Authors:** Shuaishuai Cui, Zhaoyang Jin, Tonglin Yu, Cunxin Guo, Yujian He, Yuhe Kan, Liang Yan, Li Wu

**Affiliations:** 1 School of Chemical Sciences, University of Chinese Academy of Sciences, Beijing 100049, China; cuishuaishuai22@mails.ucas.ac.cn (S.C.); jinzhaoyang20@mails.ucas.ac.cn (Z.J.); yutonglin22@mails.ucas.ac.cn (T.Y.); guocunxin22@mails.ucas.ac.cn (C.G.); yujianhe@hotmail.com (Y.H.); 2 School of Future Technology, University of Chinese Academy of Sciences, Beijing 100049, China; 3 College of Biology and Oceanography, Weifang University, Weifang 261061, China

**Keywords:** glycosylation, D-amino acid, peptide, enzymatic degradation

## Abstract

The accumulation of D-amino acid-containing peptides is associated with age-related diseases such as Alzheimer’s disease and cataracts, while glycosylation is an important modification of proteins and plays a key role in improving the physicochemical properties of peptides and facilitating their regulation in biological systems. This study investigates the effects of glycosylation position, glycan number, and monosaccharide structure on the conformation and enzymatic degradation of D-amino acid-containing peptides, using KYNEtWRSED (5-t) as a model peptide and six monosaccharides as model glycans. The results demonstrated that glycosylation inhibited the enzymatic degradation of 5-t in the presence of most serine-like proteases. However, in the presence of chymotrypsin, glycosylation with modified monosaccharides (except for β-D-GalNAc) promoted the degradation of 5-t. Furthermore, glycosylation had no effect on the cleavage site of 5-t. Molecular docking analysis revealed that the hydrogen bonding and electrostatic interactions between the glycopeptide and chymotrypsin were markedly strengthened, likely serving as a key determinant of the enzymatic effects. Collectively, these findings highlight the potential of glycosylation to enhance the therapeutic and biomedical applications of D-amino acid-containing peptides in disease treatment and drug design.

## 1. Introduction

Peptides and proteins are fundamental components of living organisms. Among them, peptide bond cleavage plays a critical role in regulating physiological functions in living organisms [1,2,3]. Although protein hydrolases can efficiently and rapidly hydrolyze peptide bonds under mild conditions [4], their substrate range is largely limited to genetically encoded amino acid sequences [5]. When L-amino acids are replaced by the corresponding D-amino acids, the conformations of peptides or proteins change, causing difficulties in recognition and timely clearance by proteases [6,7]. Over time, their accumulation in the body may lead to diseases such as Alzheimer’s disease, cataracts, macular degeneration, and atherosclerosis [8,9,10]. Therefore, effective regulation of peptides altered by D-amino acids insertions is crucial for health maintenance.

In the current study, various approaches were used to degrade peptides containing non-natural amino acids (D-amino acids), focusing primarily on metal ion complexation (Zn [11], Ni [12,13], Zr [14,15], Cu [16], Sc [17,18], etc.,) and chemical methods [19,20,21,22] for selective hydrolysis of peptide bonds. Most of these methods rely on heavy metal ions and organic solvents, which limits their applicability in physiological environments. In contrast, enzymatic digestion by protein hydrolases under physiological conditions does not disrupt normal physiological activities. The insertion of D-amino acids inhibits the enzymatic digestion of peptides and proteins, making it challenging to eliminate D-amino acid-containing peptides via enzymatic digestion. Differently modified peptides may have varying effects on the enzymatic reaction. Covalent attachment of bioactive molecules to peptides, such as PEGylation [23,24,25,26], N-terminal acetylation [27,28], glycosylation [29,30], etc., has been reported to modulate the activity and structural characteristics of peptides. Our previous study demonstrated that the conjugation of mini-PEG significantly promoted the degradation of D-amino acid-containing peptides [31]. Unlike in vitro PEG modifications, glycosylation occurs widely in physiological environments in vivo, influencing the properties and functions of peptides [32,33,34]. For normal peptides and proteins, glycosylation typically enhances target specificity [35], improves biofilm permeability [36], increases metabolic stability, and reduces clearance [37], etc. For example, Su-Wei Dong found that glycopeptides with α- or β-heterocapsids exhibited the opposite gelation abilities when the glycan portion contained axial hydroxyl groups, likely due to the aromatic CH-π bond promoting glycopeptide self-assembly [38]. Similarly, Zhao Xia [39] utilized bee venom peptide as a model and demonstrated that the glycosylation approach improves the proteolytic stability and cell selectivity of HYL-14. The chirality of monosaccharides also exhibited differential effects: α-linked D-monosaccharides enhanced the antitumor activity of HYL, while L-monosaccharides improved its tumor cell selectivity. While these studies provide valuable insights, they primarily focus on glycosylation’s effects on natural peptides. To date, the impact of glycosylation on peptides containing D-amino acids, which have unique structural and functional properties, remains largely unexplored. Therefore, this study aims to investigate glycosylation’s effect on the enzymatic degradation of D-amino acid-modified peptides, building on prior research.

In our previous study [6], the template peptide KYNETWRSED, derived from aldo-keto reductase [40] (a metabolic enzyme associated with cancer), was investigated. It was found that substituting Threonine (Thr) at position five and Tryptophan (Trp) at position six with the corresponding D-amino acids caused the most substantial inhibition of enzymatic degradation, suggesting that these two amino acids play a critical role in the peptide’s enzymatic cleavage site. Additionally, since Threonine and serine are often subject to O-glycosylation, we selected a peptide containing D-Thr at position five (KYNEtWRSED, 5-t) as the template peptide to investigate the effects of glycosylation on the structure and enzymatic activity of D-amino acid-containing peptides using various monosaccharides. Here, we examined both the influence of monosaccharide structures, comparing five-membered and six-membered rings, and the impact of monosaccharide chirality (e.g., α-Glucose vs. β-Glucose, L-Ribose vs. D-Ribose). These findings provide practical insights into controlling pathogenic D-amino acid-containing peptides or proteins.

## 2. Results

### 2.1. Design and Synthesis of Glycosylated Peptides

**Figure 1:**
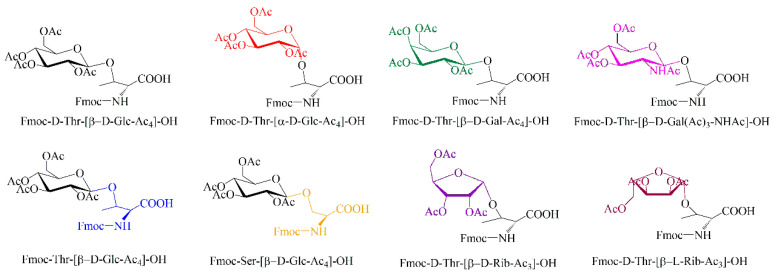
Glycosylated amino acids.

To investigate the effects of glycosylation, we designed and synthesized a series of modified peptides using eight different glycosylated amino acids (Figure 1). β-D-Glucose (β-D-Glc) was initially selected for the glycosylation of Thr at site five (Thr5) and Ser at site eight (Ser8) to evaluate the effects of glycosylation site and the number of monosaccharides on the normal peptide (all-_L_) and the D-amino acid-containing peptide KYNEtWRSED (5-t). Subsequently, five additional monosaccharides, including α-D-Glucose (α-D-Glc), β-D-Galactose (β-D-Gal), β-D-acetyl-Galactosamine (β-D-GalNAc), β-D-Ribose (β-D-Rib), and β-L-Ribose (β-L-Rib) were selected for glycosylation at Thr5 to investigate the impact of monosaccharide structure on 5-t. Among these factors, the influence of monosaccharide chirality, in addition to amino acid chirality, is of particular interest.

**Figure 2:**
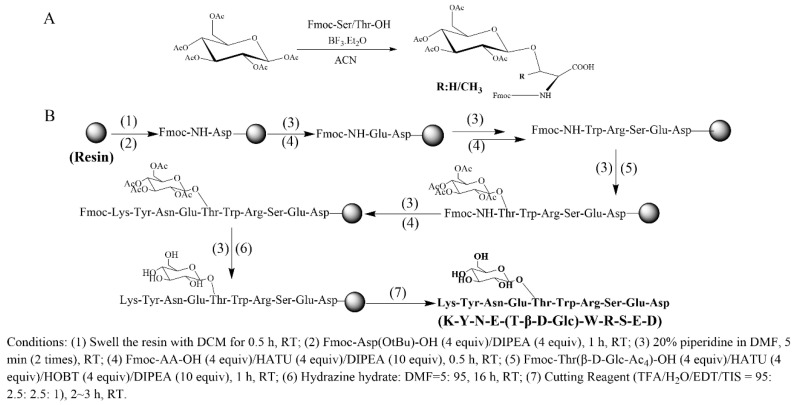
Schematic diagram of the synthesis of glycoamino acids (**A**) and glycosylated peptides (**B**).

According to the previous report [39], eight glycoamino acids were synthesized from the Fmoc-Thr-OH/Fmoc-Ser-OH and the corresponding monosaccharides (Figure 2A). The glycosylated peptides were prepared using a solid-phase peptide synthesis procedure with Rink Amide MBHA resin as the solid support [39] (Figure 2B). The coupling of glycoamino acids was performed using a method similar to that of Fmoc-Thr-OH/Fmoc-Ser-OH. The measured molecular weight of each peptide was consistent with their theoretically calculated value (Table S2).

### 2.2. Effect of Monosaccharides on Peptide Structure

Based on structural variability, eight glycosylated amino acids (Figure 1) were selected, including L-Thr-(β-D-Glc), D-Thr-(β-D-Glc), L-Ser-(β-D-Glc), D-Thr-(α-D-Glc), D-Thr-(β-D-Gal), D-Thr-(β-D-GalNAc), D-Thr-(β-D-Rib), and D-Thr-(β-L-Rib). These glycosylated amino acids were incorporated at Thr5 and Ser8 of the sequence KYNETWRSED, yielding a series of related glycopeptides (Table 1).

**Figure 3:**
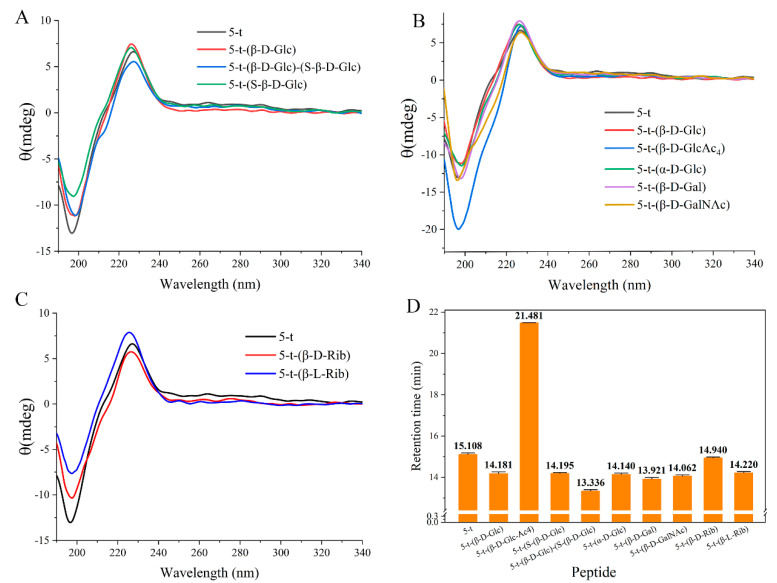
Effect of glycosylation sites, number (**A**), different monosaccharides (**B**), and monosaccharide chirality (**C**) on the CD spectrum of 5-t. Result of glycosylation modifications on the retention time of peptides in RP-HPLC (**D**).

The results of the structural studies revealed that the conformation of the monosaccharide, the number of monosaccharides, and the modification sites have a minimal impact on the overall structure of the target polypeptides. As shown in Figure 3A, despite variations in the number and positioning of monosaccharides attached to the peptides, which resulted in differences in circular dichroism (CD) spectra absorption peaks, peptides 5-t-(β-D-Glc), 5-t-(S-β-D-Glc), and 5-t-(β-D-Glc)-(S-β-D-Glc) exhibited identical random coil conformations to peptide 5-t. Additionally, no significant conformational changes were observed in the peptides modified with various structural monosaccharides (α-D-Glc, β-D-Glc, β-D-Glc-Ac4, β-D-Gal, and β-D-GalNAc) compared to 5-t (Figure 3B). Furthermore, CD spectra of 5-t-(β-D-Rib) and 5-t-(β-L-Rib) demonstrated that the chirality of the monosaccharides had no significant effect on the randomly coiled structure of the peptides, although the L configuration exerted a stronger effect on the local conformation of 5-t compared to the D configuration (Figure 3C). It is noteworthy that 5-t-(β-D-Glc-Ac_4_), which contains four acetyl groups, exhibited a significantly enhanced intensity of the negative absorption peak at 197 nm (Figure 3B), which may be attributed to the pronounced hydrophobicity of the acetyl groups.

A comparison of the retention times of glycosylated peptides via high-performance liquid chromatography (HPLC) (Figure 3D) revealed that 5-t-(β-D-Glc-Ac_4_) had a significantly longer retention time (21.481 min) than 5-t, which was caused by its enhanced hydrophobicity. Modifications with other structural monosaccharides and an increase in the number of monosaccharides significantly enhanced the polarity of the peptide, as evidenced by the notably shortened retention times of glycosylated peptides compared to 5-t.

### 2.3. Effect of Glycosylation on Enzymolysis Kinetics of Peptides

**Figure 4:**
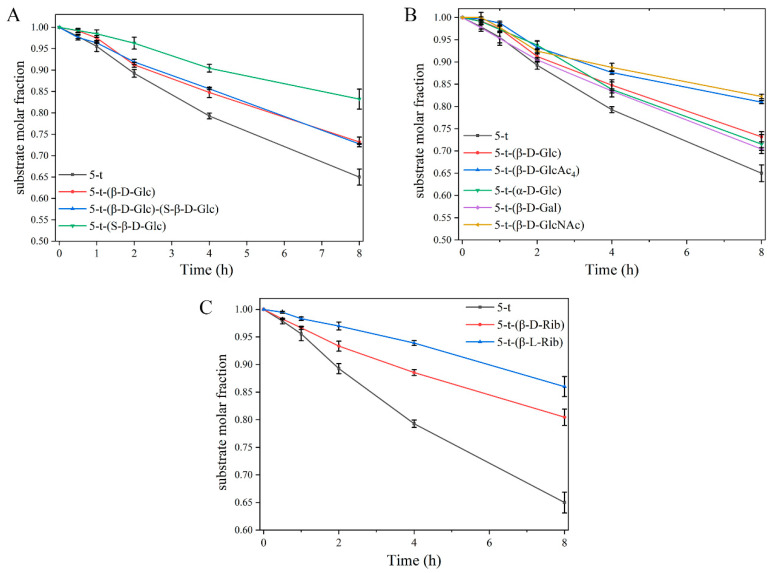
Comparison of substrate decay curves of peptides before and after glycosylation under the action of PROK. Glycosylation site and number (**A**), different monosaccharides (**B**), and monosaccharide chirality (**C**).

Building on our previous findings [6], the introduction of the corresponding D-amino acids at the enzymatic hydrolysis site significantly inhibited PROK hydrolysis. This study further demonstrates that glycosylation of D-amino acid-containing peptides exerts varying degrees of inhibition on enzymatic hydrolysis, depending on the types of monosaccharides introduced. This phenomenon was consistent with the effects of glycosylation observed in normal peptides (Figure S2). The observed inhibition could be attributed to the site-blocking effect of the modified monosaccharides, which interfered with the interaction between the protease active site and the peptide [41]. The data showed that structural modifications with different monosaccharides inhibited the enzymatic degradation of peptide 5-t by PROK to varying extents (Figure 4A). Additionally, Figure 4B showed that the substrate residue of 5-t-(S-β-D-Glc) was higher than that of 5-t-(β-D-Glc), indicating that glycosylation at Ser8 had a stronger inhibitory effect on the enzymatic degradation of 5-t compared to Thr5. The substrate decay curves for peptides 5-t-(β-D-Glc)-(S-β-D-Glc) and 5-t-(β-D-Glc) were nearly identical, suggesting that glycosylation at Thr5 plays a pivotal role in regulating the enzymatic degradation of peptide 5-t. Furthermore, analysis of substrate decay curves for 5-t-(β-D-Rib) and 5-t-(β-L-Rib) revealed that the chiral monosaccharide L-ribose had a more pronounced inhibitory effect on peptide enzymatic degradation compared to D-ribose (Figure 4C).

**Figure 5:**
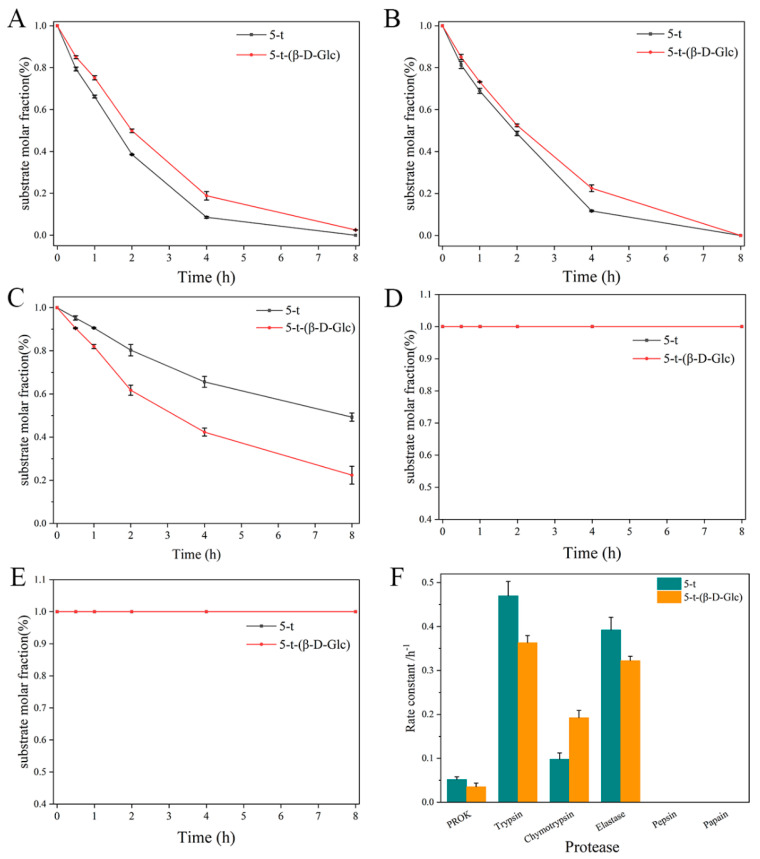
Comparison of the substrate decay curves of 5-t and 5-t-(β-D-Glc) in the presence of trypsin (**A**), elastase (**B**), chymotrypsin (**C**), pepsin (**D**), and papain (**E**), and the substrate decay rate constants (**F**).

Considering that PROK is a nonspecific serine protease commonly used for general protein digestion, glycosylation did not facilitate enzymatic digestion of the D-amino acid-containing peptides in its presence. In addition to PROK, many proteases, such as trypsin, chymotrypsin, elastase, pepsin, and papain, exhibit superior protein-degrading properties. Therefore, we further investigated the enzymatic degradation of glycopeptides in the presence of these proteases. 5-t-(β-D-Glc) and 5-t were used as substrates, and the enzymatic digestion results are shown in Figure 5 and Figure S4. The enzymatic digestion rate of 5-t-(β-D-Glc) was slightly slower than that of 5-t under the action of trypsin and elastase (Figure 5A,B), although the overall digestion efficiency did not change. After 8 h of incubation with trypsin and elastase, both 5-t-(β-D-Glc) and 5-t were degraded to the same extent. In contrast to trypsin and elastase, chymotrypsin, in combination with glycosylation, significantly enhanced the degradation of 5-t-(β-D-Glc) (Figure 5C). Additionally, neither 5-t nor 5-t-(β-D-Glc) underwent enzymatic degradation in the presence of pepsin or papain (Figure 5D,E). According to the substrate decay rate constants in Figure 5F, glycosylation inhibited the enzymatic digestion of 5-t by trypsin and elastase. In contrast, glycosylation enhanced the enzymatic digestion of 5-t in the presence of chymotrypsin.

**Figure 6:**
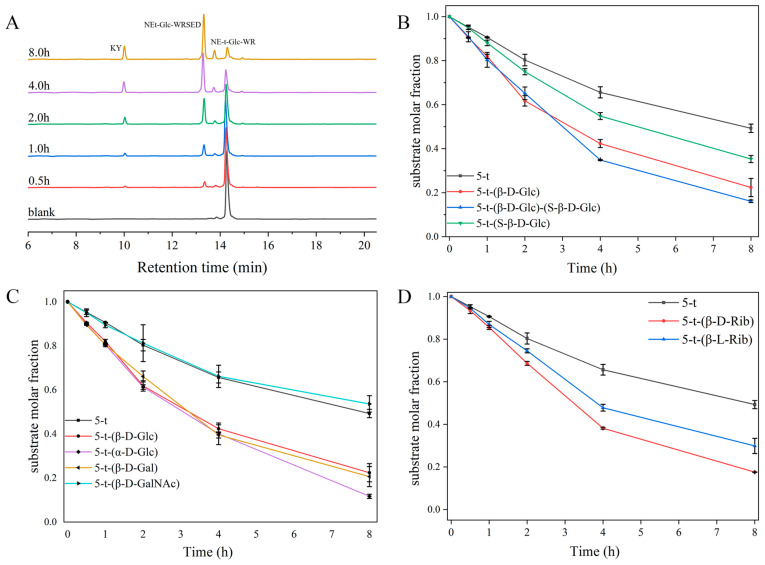
HPLC analysis of enzyme digestion results of 5-t-(β-D-Glc) (**A**) in the presence of chymotrypsin, and the substrate decay curves of glycopeptides (**B**–**D**). Peptides were dissolved in HEPES buffer solution (50 mM, pH = 7.4) at a final concentration of 0.5 mg/mL, and 0.5 mg/mL of chymotrypsin was added to the above solution. In which the peptide: protease (ω:ω) = 40:1. Incubation was performed at 37 °C. Each experiment was performed three times.

**Figure 7:**
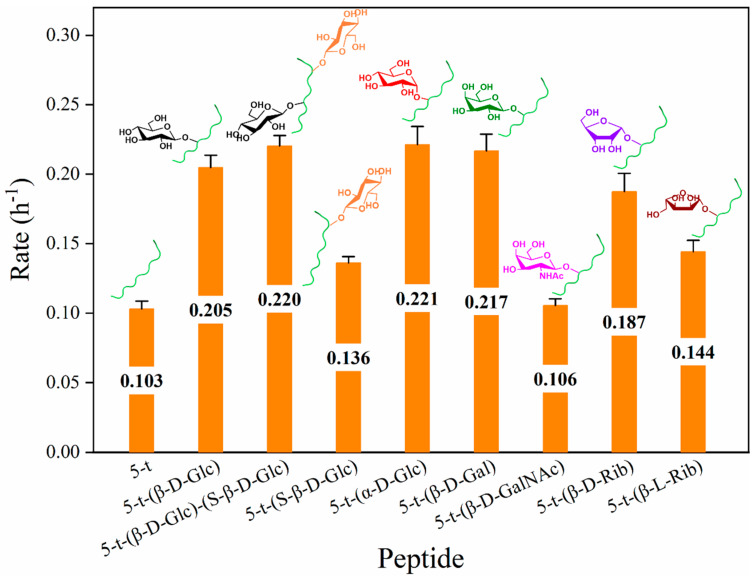
Comparison of the substrate decay rate constants of 5−t and related glycopeptides in the presence of chymotrypsin.

Building on the enzymatic promotion of peptide 5-t-(β-D-Glc) observed in the presence of chymotrypsin (Figure 6A), we next examined the effects of different glycosylation modifications on the enzymatic degradation of peptide 5-t. Combined with HPLC profiles, it was evident that all glycosylated peptides, except 5-t-(β-D-GalNAc), exhibited some degree of enzymatic promotion in the presence of chymotrypsin. According to the substrate decay curves (Figure 6B), after 8 h of enzymatic digestion with chymotrypsin, peptides 5-t-(β-D-Glc), 5-t-(S-β-D-Glc), and 5-t-(β-D-Glc)-(S-β-D-Glc) exhibited less substrate residue than 5-t. A comparison of substrate decay rate constants (Figure 7) revealed that the enzymatic degradation of 5-t was enhanced by two-site glycosylation (0.220 h⁻^1^ for 5-t-(β-D-Glc)-(S-β-D-Glc)) compared to single-site glycosylation (0.205 h⁻^1^ for 5-t-(β-D-Glc) and 0.136 h⁻^1^ for 5-t-(S-β-D-Glc)). Additionally, glycosylation at Thr5 was more influential than glycosylation at Ser8 in promoting enzymatic degradation. Under the same enzymatic conditions, analysis of the substrate decay curves and rate constants (Figure 6C and Figure 7) indicated that monosaccharide modifications with different structures had slightly varying effects on the enzymatic promotion of peptide 5-t. In particular, α-D-Glc modification had a more pronounced effect on the enzymatic hydrolysis of 5-t compared to β-D-Glc and β-D-Gal modifications. For 5-t-(β-D-GalNAc), the results were similar to those of 5-t, possibly due to the greater steric hindrance of β-D-GalNAc compared to β-D-Glc, β-D-Gal, and β-D-Glc. Finally, an examination of the impact of monosaccharide chirality (Figure 6D and Figure 7) revealed that both 5-t-(β-D-Rib) and 5-t-(β-L-Rib) were digested more efficiently than 5-t, indicating that D- and L-Rib enhance the enzymatic digestion of peptides. Among them, D-Rib (0.187 h⁻^1^) was significantly more effective than L-Rib (0.144 h⁻^1^) in promoting the digestion of 5-t. In general, modifications to the site, number, structure, and chirality of monosaccharides exert varying effects on the enzymatic degradation of 5-t by chymotrypsin.

### 2.4. Effect of Glycosylation on Enzymatic Cleavage Site of 5-t

To further investigate how glycosylation promotes the enzymatic digestion of D-amino acid-containing peptides, we analyzed the enzymatic cleavage sites of the glycosylated peptides using LC-MS (Figures S6–S14). Previous reports showed that the enzymatic fragments of 5-t were mainly KY, NE-t-WR, NE-t-WRSED, and SED, with the corresponding cleavage sites at Tyr2-Asn3 and Arg7-Ser8 (Table 2). The enzymatic fragments of 5-t-(β-D-Glc), 5-t-(β-D-Glc)-(S-β-D-Glc), 5-t-(α-D-Glc), 5-t-(β-D-Gal), and 5-t-(β-D-GalNAc) were identical to those of 5-t, indicating that the cleavage sites (Tyr2-Asn3 and Arg7-Ser8) remained unchanged. Subsequent analysis of the enzymatic fragments of 5-t-(S-β-D-Glc), 5-t-(β-D-Rib), and 5-t-(β-L-Rib) revealed that, although their overall enzymatic fragments were identical to those of 5-t, all three peptides contained an additional fragment: KYNE-t-WR, KYNE-(t-β-D-Rib)-WR, and KYNE-(t-β-L-Rib)-WR, respectively. However, the appearance of this fragment did not alter the enzymatic cleavage sites of the peptide, which was primarily attributed to incomplete cleavage at Tyr2-Asn3. These findings indicate that glycosylation does not affect the enzymatic cleavage sites of 5-t.

## 3. Discussion

**Figure 8:**
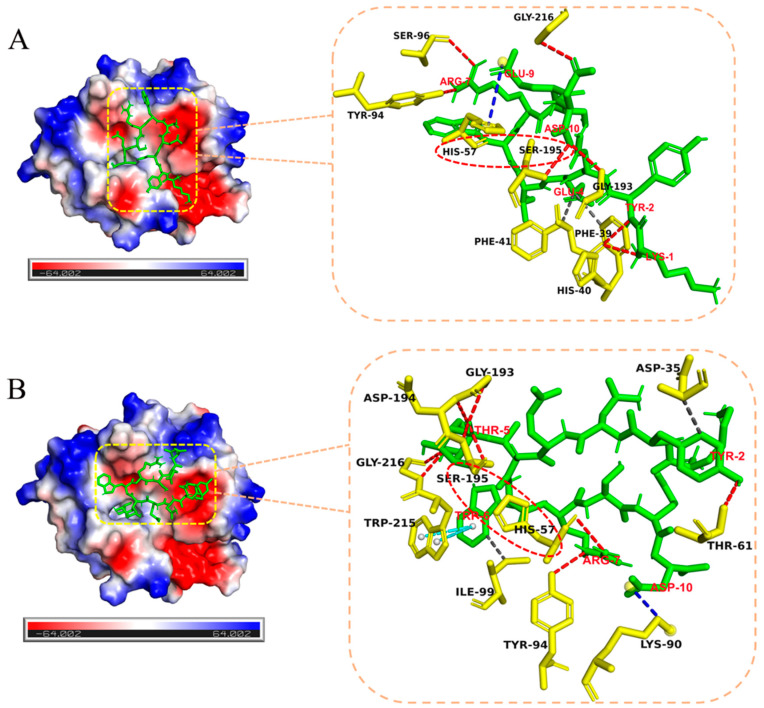
Computational simulation of the interaction of 5-t (**A**) and 5-t-(β-D-Glc) (**B**) with chymotrypsin. The green segment is the ligand, and the yellow part is the residue in the receptor. The red characters in the figure belong to the ligand amino acids, and the black characters belong to the receptor amino acids. Hydrogen bonds (red, ---), hydrophobic interactions (gray, ---), salt bridges (blue, ---), and π-π stacking (blue-green, ---).

To understand the role of glycosylation in facilitating the enzymatic digestion of D-amino acid-containing peptides by chymotrypsin, molecular docking was performed to reveal the interactions between glycosylated peptide 5-t-(β-D-Glc) and non-glycosylated peptide 5-t with chymotrypsin, as shown in Figure 8. The active center of chymotrypsin (derived from *Bos taurus*, PDB ID: 4CHA) is located within the catalytic subunit, where residues His57, Asp102, and Ser195 are essential for its enzymatic activity. Asp102 functions as an anchor, while His57 acts as a generalized base, abstracting a proton from the hydroxyl group of the Ser195 side chain. The negatively charged oxygen from Ser195 then attacks the carbonyl carbon in the peptide bond, leading to peptide bond cleavage. Importantly, the formation of an optimal catalytic triad is crucial for efficient peptide bond cleavage. For both 5-t and 5-t-(β-D-Glc), His57 and Ser195 in the active site of chymotrypsin were involved in enzymatic cleavage, with no differences observed in the construction of the catalytic triad.

Thus, the facilitation of enzymatic degradation may be attributed to the interaction forces between the substrate and chymotrypsin, as summarized in Tables S3 and S4. For peptide 5-t-(β-D-Glc) (Table S4 and Figure 8B), His57, Thr61, Tyr94, Gly193, Asp194, Ser195, and Gly216 in chymotrypsin formed precise hydrogen bonds with Arg7, Tyr2, Thr5 of the substrate. Additional hydrogen bonds were observed on the side chain monosaccharide of Thr5, primarily involving the hydroxyl group at position three and the hydroxymethyl group at position six of the monosaccharide. Additionally, two sets of π-π stacking interactions and salt bridges between Trp215 and Lys90 in chymotrypsin and Trp6 and Asp10 in 5-t-(β-D-Glc) enhanced electrostatic interactions, bringing the peptide and protease into closer proximity. The hydrophobic interactions between Asp35 and Ile99 in chymotrypsin and Tyr2 and Trp6 in 5-t-(β-D-Glc), respectively, likely facilitated the formation of transition-state structures required for enzymatic hydrolysis. In contrast, 5-t exhibited the same number of salt bridges and hydrophobic interactions with chymotrypsin. However, its overall hydrogen bonding was weaker than that of 5-t-(β-D-Glc) (Table S3). Moreover, the offset face-to-face π-π stacking interaction observed between 5-t-(β-D-Glc) and chymotrypsin was absent in 5-t.

In comparison, the inhibition of enzymatic digestion induced by glycosylation in the presence of PROK (from *Parengyodontium album*, PDB ID: 2ID8) was also analyzed using computational simulations. The results showed that for 5-t, only Ser224 in the catalytic triad of PROK was active, whereas for 5-t-(β-D-Glc), only His69 formed a binding interaction (Figure S15). Moreover, according to the mechanism of the catalytic triad, Ser224, acting as a nucleophile, was significantly more critical than His69, which acts as a general base. Therefore, enzymatic cleavage was inhibited due to the absence of Ser224 participation, resulting in a stronger inhibitory effect for 5-t-(β-D-Glc) compared to 5-t.

## 4. Experimental Section

### 4.1. Materials

All reagents and solvents were purchased from Wuxi Asiapeptide Biotechnology Co., Ltd. (Wuxi, China), Shaoyuan Reagent Co., Ltd. (Shanghai, China), Energy Chemical Co., Ltd. (Shanghai, China), Dipper Biotechnology Co., Ltd. (Shanghai, China), Wokai Biotechnology Co., Ltd. (Shanghai, China), Solarbio Science & Technology Co., Ltd. (Beijing, China), Psaitong Co., Ltd. (Beijing, China), Aladdin Biochemical Technology Co., Ltd. (Shanghai, China), Merida Technology Co., Ltd. (Beijing, China), and Sinopharm Chemical Reagent Co., Ltd. (Beijing, China). 2-CTC resin (0.96 mmol/g loading) was purchased from Glbiochem Co., Ltd. (Shanghai, China). All other commercially available reagents and solvents could be used directly without further purification.

### 4.2. Synthesis of Glycosylated Amino Acids

A mixture of pentaacetate monosaccharide (1 equiv) and Fmoc-Ser/Thr-OH (1.5 equiv) was dissolved with acetonitrile, followed by the addition of BF_3_·OEt_2_ (2.5 equiv). The reaction mixture was stirred at room temperature under argon for 24 h. Subsequently, the reaction solution was collected, the solvent was removed under reduced pressure, and the resulting residue was diluted with EtOAc and then washed with water. The organic layer was dried with Na_2_SO_4_ and filtered, and the filtrate was concentrated under reduced pressure. The resulting yellow oil was purified by flash chromatography on a silica gel column (Hex/EtOAc/AcOH = 4:1:0.5 → 3:1:0.4 → 2:1:0.3) to afford the final product as a white powder.

### 4.3. Peptide Synthesis

The peptides were synthesized by peptide solid-phase synthesis (SPPS) both before and after the glycosylation modification. 0.025 mmol of 2-2-chlorotrityl chloride (2-CTC) resin (0.97 mmol/g) was swollen in dichloromethane (DCM, 5 mL) in a peptide solid-phase synthesis tube for 0.5 h. After removing DCM, 0.1 mmol of Fmoc-AA-OH was added and dissolved in a small amount of dimethylformamide (DMF), followed by the addition of 0.25 mmol of N, N-diisopropylethylamine (DIPEA). Finally, the reaction mixture was shaken at room temperature for 1 h. The solvent was filtered off, and the resin was washed sequentially with DMF (3×, 5 mL), DCM (3×, 5 mL), and DMF (3×, 5 mL). The resin was treated with 3 mL of 20% piperidine/DMF solution and shaken for 5 min, followed by filtering to remove the solvent. Another 3 mL of 20% piperidine/DMF solution was added and shaken for 15 min. The resin was then washed with sequentially DMF (3×, 5 mL), DCM (3×, 5 mL), and DMF (3×, 5 mL). In a small glass vial, 0.1 mmol of Fmoc-AA-OH, 0.1 mmol of 2-(7-azabenzotriazol-1-yl)-N, N, N’, N’-tetramethyluronium hexafluorophosphate (HATU), and 0.25 mmol of DIPEA were dissolved in a small amount of DMF and added to the resin. The reaction was shaken for 0.5 h at room temperature, and the resin was sequentially washed with DMF (3×, 5 mL), DCM (3×, 5 mL), and DMF (3×, 5 mL). The deprotection, washing, and coupling steps were repeated until all amino acids were sequentially attached to the peptide chain. After synthesis was completed, 4 mL of cutting reagent (TFA:H_2_O:EDT:TIS = 94:2.5:2.5:1.0) was added, and the reaction was shaken for 2–3 h at room temperature to cleave the peptides from the resin. The filtrate was collected and dried under nitrogen. The extract was precipitated with some ice ether, centrifuged at 3000 r/min for 3 min (three times), and the solvent was discarded to yield the crude peptide. For glycosylated amino acids, 0.025 mmol of glycosylated amino acid, 0.1 mmol of HATU, 0.1 mmol of HOAt, 0.25 mmol of DIEA, and 2 mL of DMF were mixed and added to the resin. The reaction was shaken at room temperature for 1.0 h. The resin was subsequently washed sequentially with DMF (3×, 5 mL), DCM (3×, 5 mL), and DMF (3×, 5 mL). After peptide synthesis was completed, 2 mL of hydrazine hydrate/DMF (5:95 *v*/*v*) was added to the resin to remove the acetate-protecting groups from the monosaccharides, and the resin was subsequently cleaved in the same manner to obtain the crude peptide.

### 4.4. Purification of Peptides by Reversed-Phase HPLC

All peptides were purified using an Agilent 1260 high-performance liquid chromatography (HPLC) system. Purification was performed on a ZORBAX Eclipse XDB-C18 reversed-phase column (5 μm, 9.4 × 250 mm, Agilent, Santa Clara, CA, USA) using solvent A (0.1% TFA in water) and solvent B (0.1% TFA in acetonitrile). Elution was performed at a flow rate of 1 mL/min with a gradient of 10–50% solvent B over 30 min, and detection was carried out using a UV detector at 220 nm. All purified peptide samples were freeze-dried to yield a white powder.

### 4.5. High-Resolution Mass Spectra

The synthesized peptides were confirmed using a Thermo Q Exactive Orbitrap mass spectrometer (Waltham, MA, USA). Sample peptides were dissolved in 50% ACN/H_2_O. Electrospray ionization (ESI) was used as the ionization source with the following parameters: sheath gas flow rate, 35 L/min; auxiliary gas flow rate, 250 °C; spray voltage, 3.50 kV; capillary temperature, 320 °C; auxiliary gas heater temperature, 350 °C; mobile phase, 50% MeOH/H_2_O; flow rate, 0.2 mL/min; and injection volume, 1 μL. Also, the measured peptides were mainly present in the double-charged form and rarely in the single-charged form. Therefore, the relationship between the molecular weight M_w_ of the peptide and the mass spectrometry measurement M_test_ was M_w_ = (M_test_ − M_c_) × z, where z is the number of charges and n is the number of H^+^. A comparison between the calculated molecular weight M_w_ of the peptide and the theoretical molecular weight M_theory_ of the target peptide showed that the final molecular weight matched the theoretical value, confirming the successful synthesis of the desired peptide.

### 4.6. Circular Dichroism

CD spectra were recorded at room temperature using a 2 mm quartz cuvette on a Jasco-815 spectrophotometer (Chiyoda City, Japan). The measurement parameters were as follows: Wavelength, 190–340 nm; Stepper resolution, 0.5 nm; Speed, 200 nm/min. The curve was smoothed with standard parameters. The sample concentration was 0.3 mg/mL of aqueous solution. Each resulting CD data point was the average of the three measurements.

### 4.7. Protease Resistance

The peptides were dissolved in HEPES buffer (50 mM, pH = 7.4) to a final concentration of 0.5 mg/mL. Proteinase K (PROK) was prepared in deionized water at a final concentration of 1.5 mg/mL. The peptide solutions (600 μL) were then incubated with PROK solution (15.0 μL) at 50 °C. At 0, 0.5, 1.0, 2.0, 4.0, and 8.0 h intervals, 100 μL of the digestion mixture was taken and quenched with 100 μL of 1% TFA in water. The enzymatic hydrolysis of peptides was monitored by HPLC at 215 nm. Each experiment was performed three times.

### 4.8. HPLC Analysis of the Enzymatic Digestion Results

The enzymatic digestion of all peptides in the presence of protease was analyzed using an Agilent 1260 high-performance liquid chromatography (HPLC) system. The mobile phases consisted of 0.1% TFA in water (solvent A) and 0.1% TFA in acetonitrile (solvent B). The elution rate was 1 mL/min on an Agilent TC-C18(2) reversed-phase column (5 μm, 4.6 × 250 mm) with a gradient of 10–50% solvent B for 30 min, detected with a 215 nm UV detector.

### 4.9. Kinetic Analysis

The calculation of the reaction rate typically relies on the presence of intermediate reaction products. In this study, we used the equation to calculate the kinetic data of the reaction [12].

$$y=A_{0}\times \exp\left(-k_{1}\times x\right)$$

In the above equation, y is the mole fraction of a given substance, x is the time, and $A_{0}$ represents the initial concentration of the substrate.

### 4.10. Docking Calculations

The protease complex structures used in this study were obtained from the RCSB Protein Data Bank (PDB IDs: 4CHA [42] and 2ID8 [43], https://www.rcsb.org/structure/4CHA, accessed on 17 January 2025; https://www.rcsb.org/structure/2ID8, accessed on 17 January 2025). The peptide structures were constructed using KingDrawPc_V5.0.0.89_Pro. and GaussView 5.0.9 x64. Molecular docking was performed using AutoDock Vina (autodocksuite-4.2.6.i86Windows) [44], with active sites generated in automated mode. The interactions were analyzed by https://plip-tool.biotec.tu-dresden.de/plip-web/plip/index, accessed on [45].

## 5. Conclusions

We designed and synthesized a series of monosaccharide-modified peptides using a glycosylation approach and systematically investigated the effects of different monosaccharides on the structure and enzymatic digestion of D-amino acid-containing peptides. The results demonstrated that the five monosaccharides, inserted at different sites and in varying numbers, did not alter the secondary structure of the peptides but interfered with their local conformations. Kinetic analysis of enzymatic digestion revealed that glycosylation exerted an inhibitory effect on the enzymatic degradation of peptides by most of the proteases examined in this study. However, in the presence of chymotrypsin, monosaccharide modifications, except for β-D-GalNAc, differentially promoted the enzymatic degradation of 5-t. This enzymatic facilitation may result from enhanced interactions between the glycosylated D-amino acid-containing peptides and chymotrypsin. These findings suggest that glycosylation, while enhancing the enzymatic stability of normal peptides, may contribute to the enzymatic degradation of D-amino acid-containing peptides. Our study contributes to a deeper understanding of the theoretical significance of glycosylation and lays a solid foundation for the future development of drugs to treat diseases associated with D-amino acid peptides.

## Figures and Tables

**Table 1:** Glycopeptide sequences.

Peptides	Sequence
all-_L_	KYNETWRSED
all-_L_-(T-β-D-Glc)	KYNE(T-β-D-Glc)WRSED
all-_L_-(T-β-D-Glc)-(S-β-D-Glc)	KYNE(T-β-D-Glc)WR(S-β-D-Glc)ED
5-t	KYNEtWRSED
5-t-(β-D-Glc)	KYNE(t-β-D-Glc)WRSED
5-t-(β-D-Glc)-(S-β-D-Glc)	KYNE(t-β-D-Glc)WR(S-β-D-Glc)ED
5-t-(S-β-D-Glc)	KYNEtWR(S-β-D-Glc)ED
5-t-(β-D-GlcAc_4_)	KYNE(t-β-D-Glc-Ac_4_)WRSED
5-t-(α-D-Glc)	KYNE(t-α-D-Glc)WRSED
5-t-(β-D-Gal)	KYNE(t-β-D-Gal)WRSED
5-t-(β-D-GalNAc)	KTNE(t-β-D-Gal-NAc)WRSED
5-t-(β-D-Rib)	KYNE(t-β-D-Rib)WRSED
5-t-(β-L-Rib)	KYNE(t-β-L-Rib)WRSED

Note: t denotes the D-Threonine (D-Thr).

**Table 2:** Enzymatic fragments and peptide digestion site analysis of peptides in the presence of chymotrypsin before and after glycosylation.

Peptides	Enzymatic Fragments	Cleavage Sites
5-t	KY, NE-t-WR, NE-t-WRSED, SED	Tyr2-Asn3, Arg7-Ser8
5-t-(β-D-Glc)	KY, NE-(t-β-D-Glc)-WR, NE-(t-β-D-Glc)-WRSED, SED	Tyr2-Asn3, Arg7-Ser8
5-t-(β-D-Glc)-(S-β-D-Glc)	KY, NE-(t-β-D-Glc)-WR, NE-(t-β-D-Glc)-WR-(S-β-D-Glc)-ED, (S-β-D-Glc)-ED	Tyr2-Asn3, Arg7-Ser8
5-t-(S-β-D-Glc)	KY, KYNE-t-WR, NE-t-WR, NE-t-WR-(S-β-D-Glc)-ED, (S-β-D-Glc)-ED	Tyr2-Asn3, Arg7-Ser8
5-t-(α-D-Glc)	KY, NE-(t-α-D-Glc)-WR, NE-(t-α-D-Glc)-WRSED, SED	Tyr2-Asn3, Arg7-Ser8
5-t-(β-D-Gal)	KY, NE-(t-β-D-Gal)-WR, NE-(t-β-D-Gal)-WRSED, SED	Tyr2-Asn3, Arg7-Ser8
5-t-(β-D-GalNAc)	KY, NE-(t-β-D-GalNAc)-WR, NE-(t-β-D-Gal-NAc)-WRSED, SED	Tyr2-Asn3, Arg7-Ser8
5-t-(β-D-Rib)	KY, KYNE-(t-β-D-Rib)-WR, NE-(t-β-D-Rib)-WR, NE-(t-β-D-Rib)-WRSED, SED	Tyr2-Asn3, Arg7-Ser8
5-t-(β-L-Rib)	KY, KYNE(t-β-L-Rib)-WR, NE-(t-β-L-Rib)-WR, NE-(t-β-L-Rib)-WRSED, SED	Tyr2-Asn3, Arg7-Ser8

Note: t denotes the corresponding D-Threonine (D-Thr).

## Data Availability

Data are contained within the article and Supplementary Materials.

## Supplementary Materials

The following supporting information can be downloaded at https://www.mdpi.com/article/10.3390/molecules30030441/s1, Table S1. ESI data of glycosylated amino acids; Table S2. ESI data of peptides; Figure S1. Comparison of CD (A) and retention time (B) of all-_L_, all-_L_-(T-β-D-Glc) and all-_L_-(T-β-D-Glc)-(S-β-D-Glc); Figure S2. Comparison of the enzymatic results of all-_L_(A), all-_L_-(T-β-D-Glc) (B) and all-_L_-(T-β-D-Glc)-(S-β-D-Glc) (C) under the action of PROK. Comparison of substrate decay curves of peptides before and after glycosylation under the action of PROK. (A) Normal peptide (all-_L_) (D); Figure S3. HPLC analysis of the results of enzymatic digestion of peptide 5-t before and after glycosylation modification in the presence of PROK. (A) 5-t, (B) 5-t-(β-D-Glc), (C) 5-t-(β-D-Glc)-(S-β-D-Glc), (D) 5-t-(S-β-D-Glc), (E) 5-t-(β-D-Glc-Ac4), (F) 5-t-(α-D-Glc), (G) 5-t-(β-D-Gal), (H) 5-t-(β-D-GalNAc), (I) 5-t-(β-D-Rib), (J) 5-t-(β-L-Rib); Figure S4. The enzymatic results of 5-t and 5-t-(β-D-Glc) in the presence of trypsin (A), chymotrypsin (B), elastase (C), pepsin (D), and papain (E). Peptides were dissolved in HEPES buffer solution (50 mM, pH = 7.4) at a final concentration of 0.5 mg/mL, and 0.5 mg/mL of protease solution was added to the above solution. In which the peptide: protease (ω: ω) = 40:1. Incubation was performed at 37 °C. Method of stopping enzymatic digestion after reaching reaction time: all enzymes were extinguished with 1% TFA, except for the enzymatic digestion of pepsin with 0.5 M NaHCO3 in equal volume. Each experiment was performed three times; Figure S5. HPLC results of 5-t (A), 5-t-(β-D-Gal) (B), 5-t-(β-D-Gal)-(S-β-D-Glc) (C), 5-t-(S-β-D-Glc) (D), 5-t-(α-D-Glc) (E), 5-t-(β-D-Rib) (F), 5-t-(β-L-Rib) (G), and 5-t-(β-D-GalNAc) (H) in the presence of chymotrypsin. The peptides were dissolved in HEPES buffer solution (50 mM, pH = 7.4) at a final concentration of 0.5 mg/mL, and 0.5 mg/mL of chymotrypsin was added to the above solution. In which the peptide: protease (ω: ω) = 40:1. Incubation was performed at 37 °C. Method of stopping enzymatic digestion after reaching reaction time: chymotrypsin is extinguished with 1% TFA. Each experiment was performed three times; Figure S6. Analysis of the enzymatic fragments of peptide 5-t in the presence of chymotrypsin by LC-MS (ESI). Among them, t indicates D-Threonine; Figure S7. Analysis of the enzymatic fragments of peptide 5-t-(β-D-Glc) in the presence of chymotrypsin by LC-MS (ESI). Among them, t indicates D-Threonine; Figure S8. Analysis of the enzymatic fragments of peptide 5-t-(β-D-Glc)-(S-β-D-Glc) in the presence of chymotrypsin by LC-MS (ESI). Among them, t indicates D-Threonine; Figure S9. Analysis of the enzymatic fragments of peptide 5-t-(S-β-D-Glc) in the presence of chymotrypsin by LC-MS (ESI). Among them, t indicates D-Threonine; Figure S10. Analysis of the enzymatic fragments of peptide 5-t-(α-D-Glc) in the presence of chymotrypsin by LC-MS (ESI). Among them, t indicates D-Threonine; Figure S11. Analysis of the enzymatic fragments of peptide 5-t-(β-D-Gal) in the presence of chymotrypsin by LC-MS (ESI). Among them, t indicates D-Threonine; Figure S12. Analysis of the enzymatic fragments of peptide 5-t-(β-D-GalNAc) in the presence of chymotrypsin by LC-MS (ESI). Among them, t indicates D-Threonine; Figure S13. Analysis of the enzymatic fragments of peptide 5-t-(β-D-Rib) in the presence of chymotrypsin by LC-MS (ESI). Among them, t indicates D-Threonine; Figure S14. Analysis of the enzymatic fragments of peptide 5-t-(β-L-Rib) in the presence of chymotrypsin by LC-MS (ESI). Among them, t indicates D-Threonine; Table S3. Analysis of the interaction between 5-t and chymotrypsin; Table S4. Analysis of the interaction between 5-t-(β-D-Glc) and chymotrypsin; Figure S15. Computational simulation of the interaction of 5-t (A) and 5-t-(β-D-Glc) (B) with PROK. The green segment is the ligand, and the yellow segment is the residue in the receptor. The red characters in the figure belong to the ligand amino acids, and the black characters belong to the receptor amino acids. Hydrogen bonds (red, ---), salt bridges (blue, ---), and π-π stacking (blue-green, ---); Figure S16–S28. Mass spectrometry results of the glycopeptides synthesized in the experiment as well as the glycosylated amino acids; Figure S29–S36. 1H-NMR, 13C-NMR, ESI-MS for glycoamino acids.
