# Supplementary material for: Effect of Glycosylation on the Enzymatic Degradation of D-Amino Acid-Containing Peptides
Source: Molecules. 2025 Jan 21;30(3):441. doi: 10.3390/molecules30030441 (PMC11820358; doi:10.3390/molecules30030441)
Supplement: Supplementary file 1 [file molecules-30-00441-s001.zip › molecules-3417910-supplementary.pdf]

Supplemental Information

Table S1. ESI data of glycosylated amino acids.

Amino acid	Theoretical molecular weight ($[M+Na]^+$)	Measured molecular weight ($[M+Na]^+$)	Yield
Fmoc-L-Ser[β-D-Glc(Ac_4)]-OH	680.195	680.196	26.7%
Fmoc-L-Thr[β-D-Glc(Ac_4)]-OH	694.211	694.211	25.6%
Fmoc-D-Thr[β-D-Glc(Ac_4)]-OH	694.211	694.211	23.6%
Fmoc-D-Thr[α-D-Glc(Ac_4)]-OH	694.211	694.211	23.0%
Fmoc-D-Thr[β-D-Gal(Ac_4)]-OH	694.211	694.211	25.9%
Fmoc-D-Thr[β-D-Glc(Ac_3) NAc]-OH	693.227	693.225	29.8%
Fmoc-D-Thr[β-D-Rib(Ac_3)]-OH	622.189	622.187	16.7%
Fmoc-D-Thr[β-L-Rib(Ac_3)]-OH	622.189	622.187	17.8%

Table S2. ESI data of peptides.

Peptides	Theoretical molecular weight (m/z)	Measured molecular weight (m/z)	Maximum fragment peak
all-L	1326.598	1326.594	664.297 $[M+2H]^{2+}$
all-L-(T-β-D-Glc)	1488.650	1488.648	745.324 $[M+2H]^{2+}$
all-L-(T-β-D-Glc)-(S-β-D-Glc)	1650.700	1650.700	826.350 $[M+2H]^{2+}$
5-t	1326.598	1326.596	664.298 $[M+2H]^{2+}$
5-t-(β-D-Glc)	1488.650	1488.648	745.324 $[M+2H]^{2+}$
5-t-(β-D-Glc)-(S-β-D-Glc)	1650.704	1650.702	826.351 $[M+2H]^{2+}$
5-t-(S-β-D-Glc)	1488.650	1488.648	745.324 $[M+2H]^{2+}$
5-t-(β-D-Glc-Ac_4)	1656.692	1656.690	829.345 $[M+2H]^{2+}$
5-t-(α-D-Glc)	1488.650	1488.646	745.323 $[M+2H]^{2+}$
5-t-(β-D-Gal)	1488.650	1488.648	745.324 $[M+2H]^{2+}$
5-t-(β-D-GalNAc)	1529.678	1529.674	765.837 $[M+2H]^{2+}$
5-t-(β-D-Rib)	1458.640	1458.638	730.319 $[M+2H]^{2+}$
5-t-(β-L-Rib)	1458.640	1458.636	730.318 $[M+2H]^{2+}$

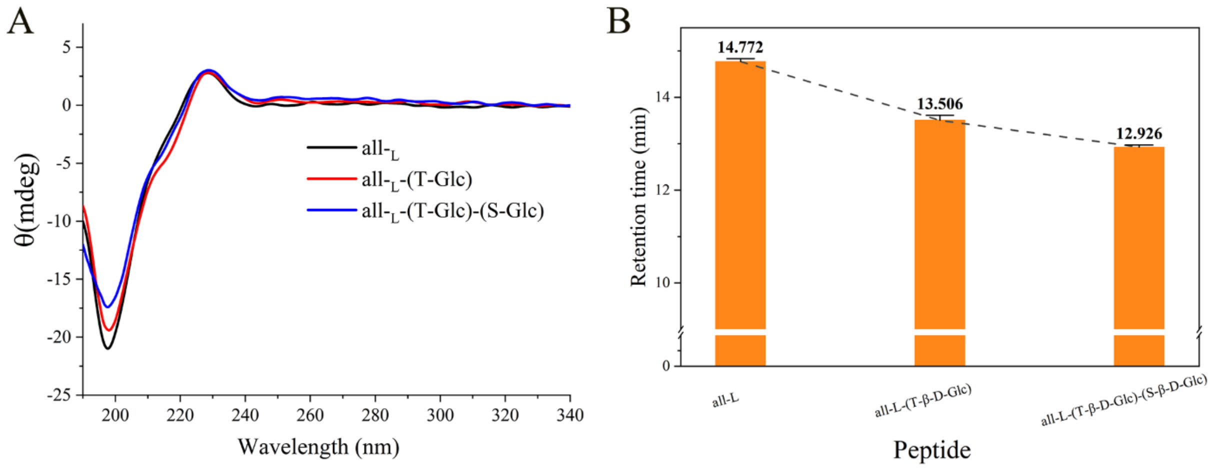

Figure S1. Comparison of CD (A) and retention time (B) of all-L, all-L-(T-β-D-Glc) and all-L-(T-β-D-Glc)-(S-β-D-Glc).

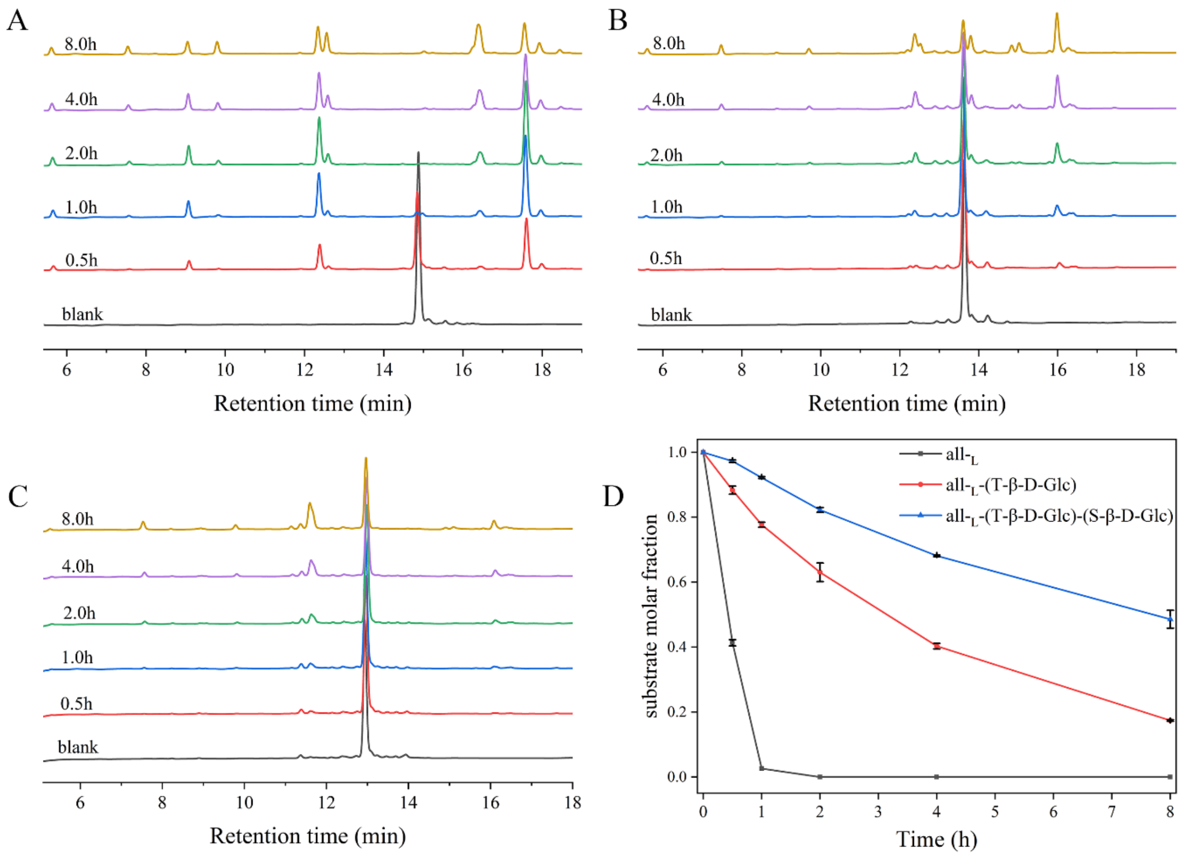

Figure S2. Comparison of the enzymatic results of all-L(A), all-L-(T-β-D-Glc) (B) and all-L-(T-β-D-Glc)-(S-β-D-Glc) (C) under the action of PROK. Comparison of substrate decay curves of peptides before and after glycosylation under the action of PROK (D).

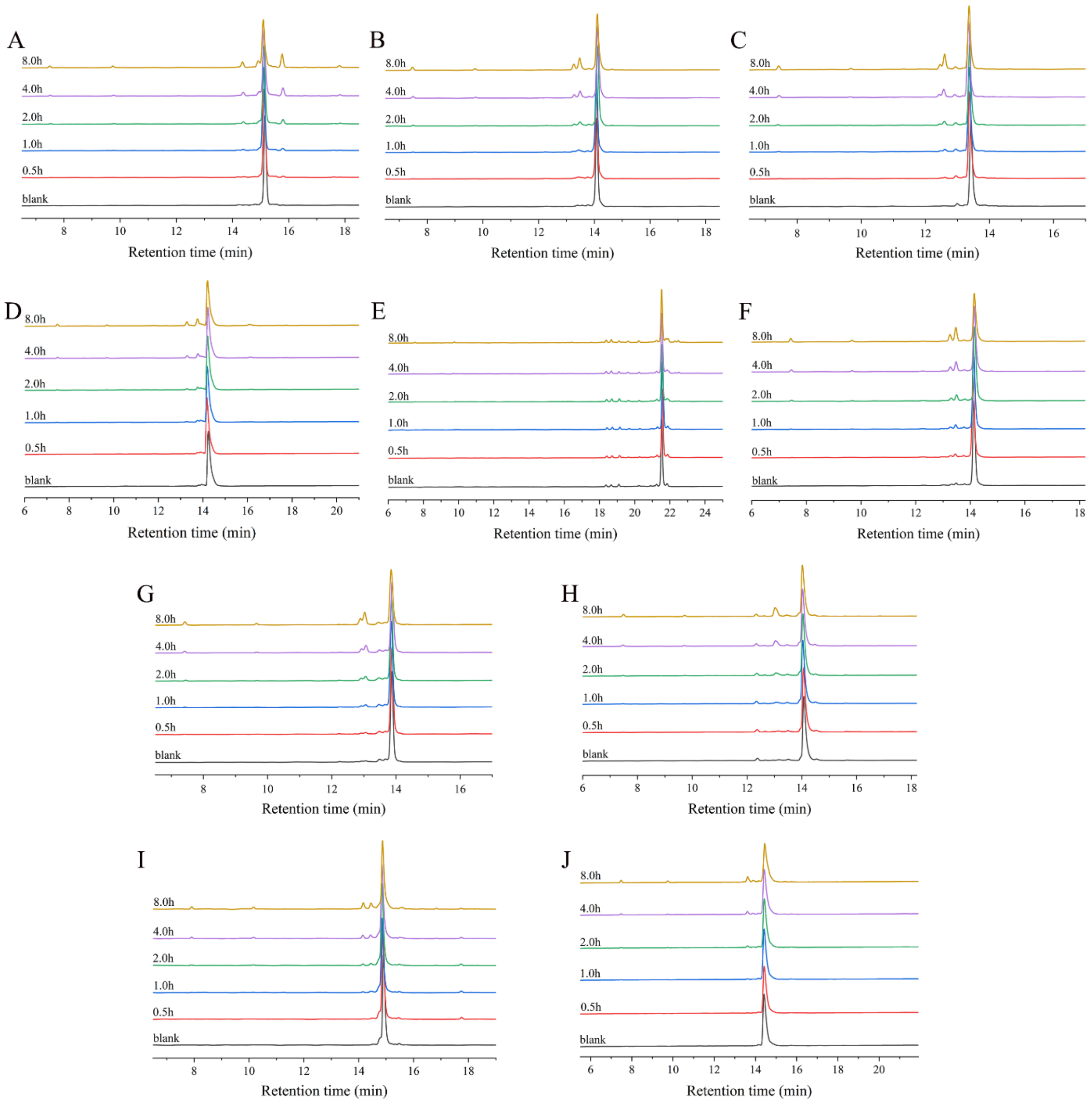

Figure S3. HPLC analysis of the results of enzymatic digestion of peptide 5-t before and after glycosylation modification in the presence of PROK. (A) 5-t, (B) 5-t-(β-D-Glc), (C) 5-t-(β-D-Glc)-(S-β-D-Glc), (D) 5-t-(S-β-D-Glc), (E) 5-t-(β-D-Glc-Ac_4), (F) 5-t-(α-D-Glc), (G) 5-t-(β-D-Gal), (H) 5-t-(β-D-GalNAc), (I) 5-t-(β-D-Rib), (J) 5-t-(β-L-Rib).

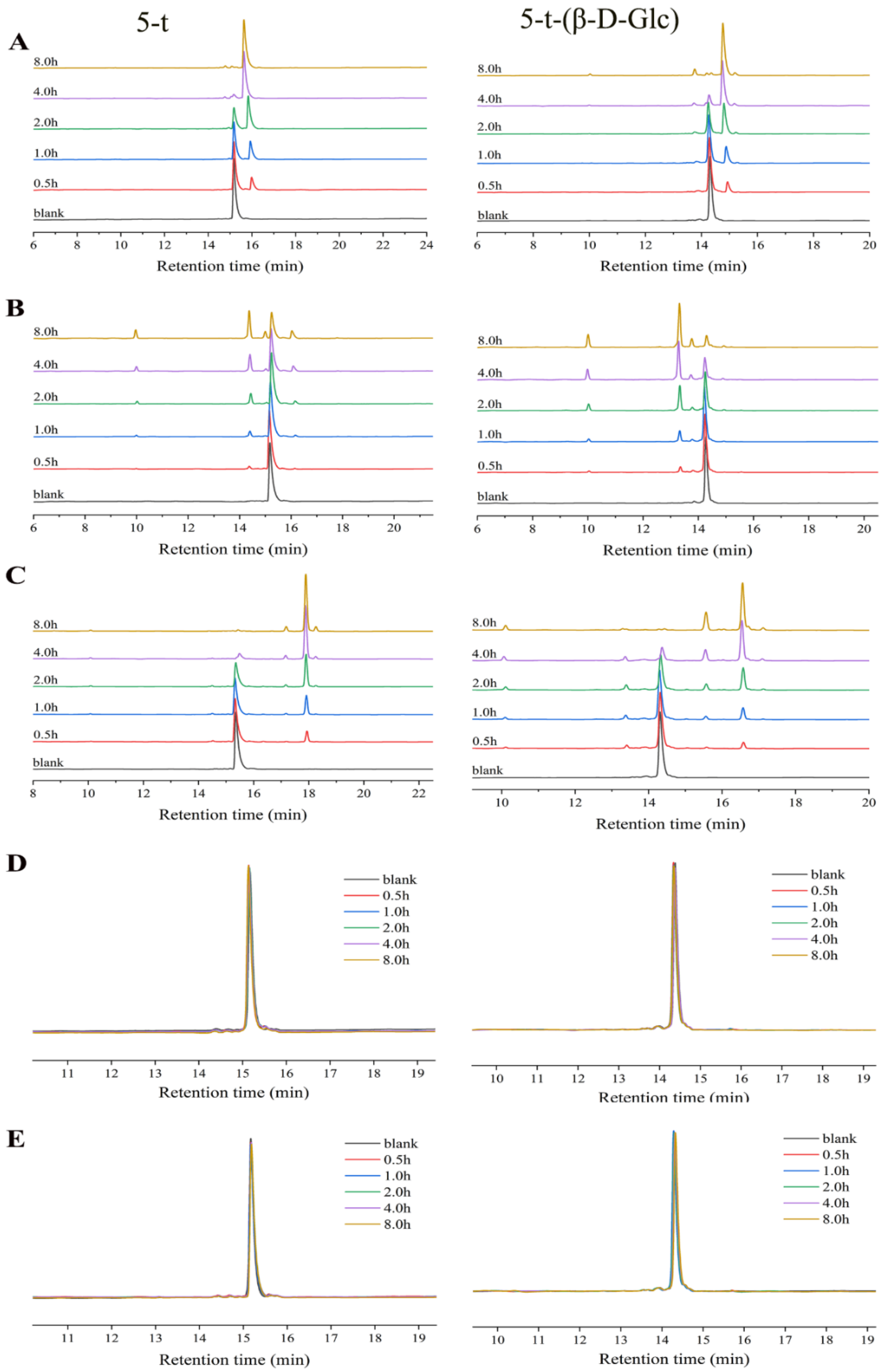

Figure S4. Enzymatic cleavage of 5-t and 5-t-(β-D-Glc) in the presence of trypsin (A), chymotrypsin (B), elastase (C), pepsin (D), and papain (E). Peptides (0.5 mg/mL) were dissolved in HEPES buffer (50 mM, pH=7.4) and incubated with protease solution (0.5 mg/mL) at a peptide-to-protease ratio of 40:1 (w/w) at 37°C. Enzymatic digestion was stopped after the reaction time using 1% TFA for all enzymes, except pepsin, which was stopped with an equal volume of 0.5 M NaHCO3. Each experiment was conducted in triplicate.

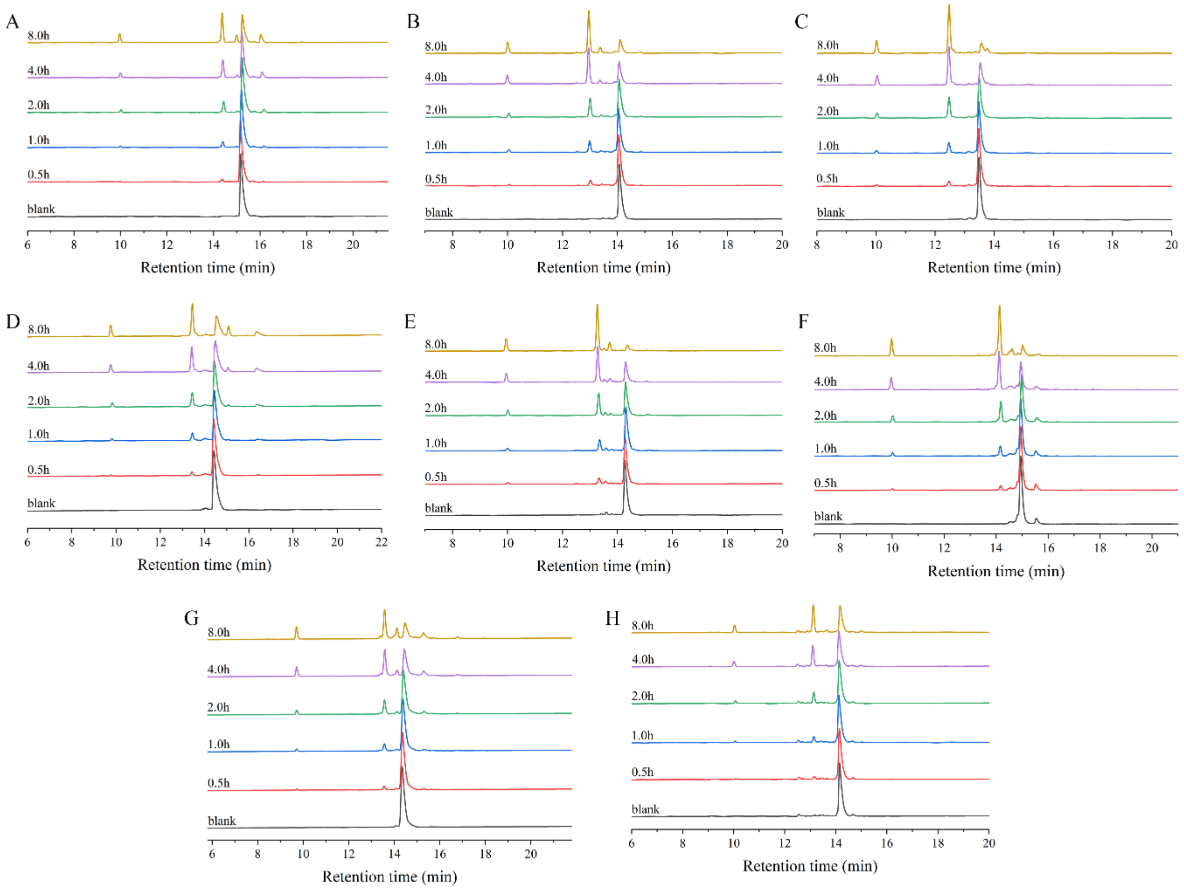

Figure S5. HPLC results of 5-t (A), 5-t-(β-D-Gal) (B), 5-t-(β-D-Gal)-(S-β-D-Glc) (C), 5-t-(S-β-D-Glc) (D), 5-t-(α-D-Glc) (E), 5-t-(β-D-Rib) (F), 5-t-(β-L-Rib) (G) and 5-t-(β-D-GalNAc) (H) in the presence of chymotrypsin. The peptides were dissolved in HEPES buffer solution (50 mM, pH=7.4) at a final concentration of 0.5 mg/mL, and 0.5 mg/mL of Chymotrypsin was added to the above solution. In which the peptide: protease (ω: ω) = 40:1. Incubation was performed at 37°C. Method of stopping enzymatic digestion after reaching reaction time: Chymotrypsin is extinguished with 1% TFA. Each experiment was performed three times.

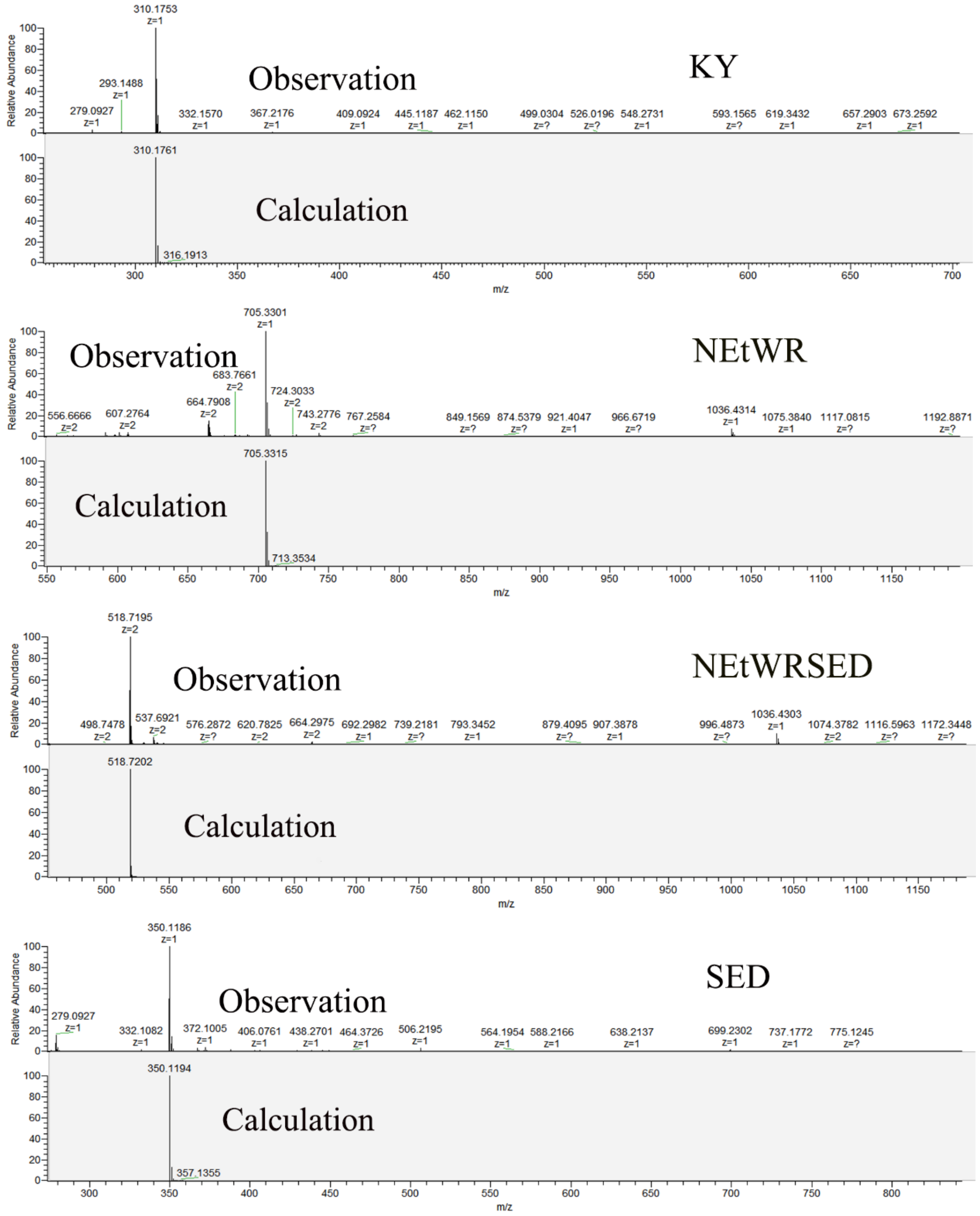

Figure S6. Analysis of the enzymatic fragments of peptide 5-t in the presence of Chymotrypsin by LC-MS (ESI). Among them, t indicates D-Threonine.

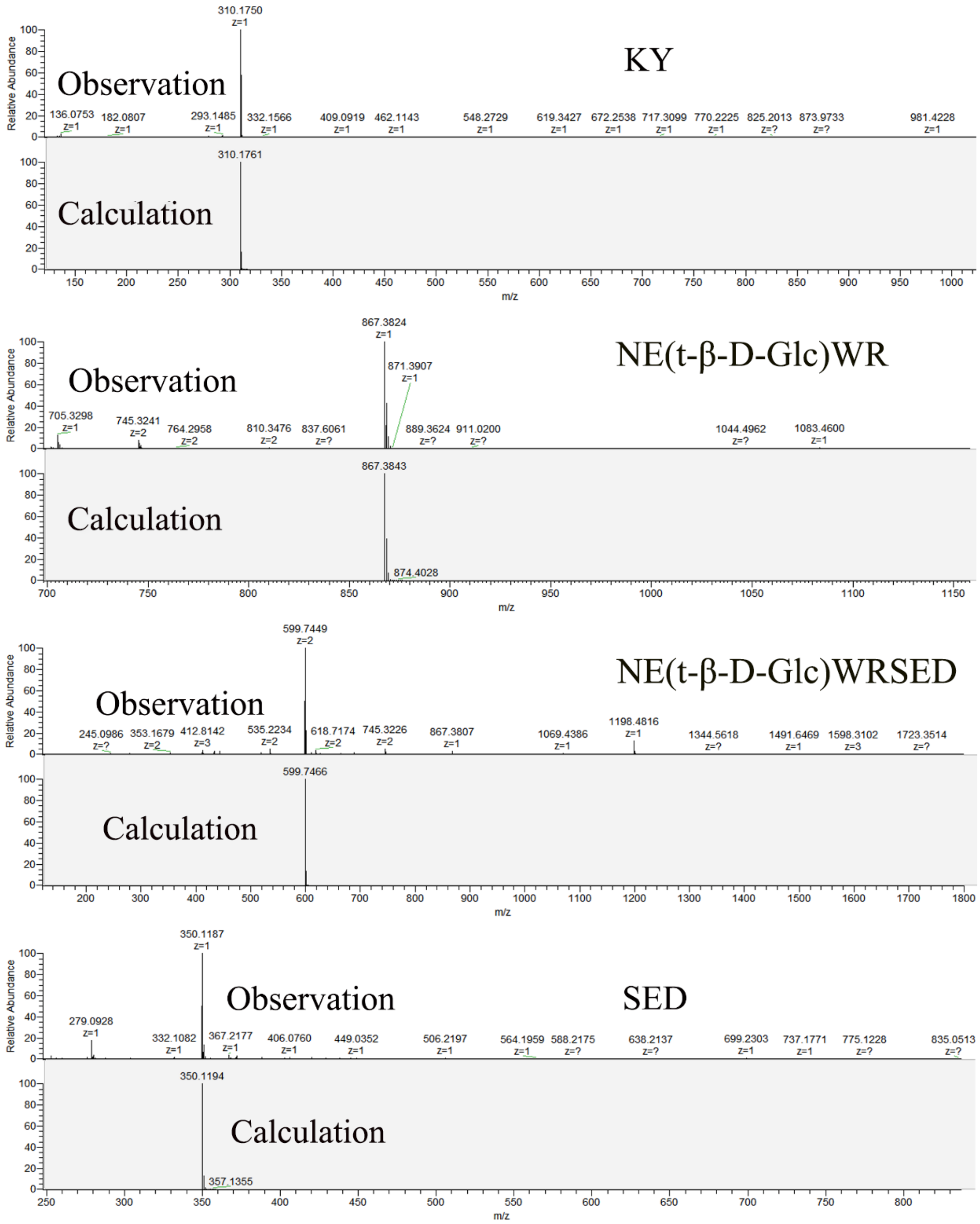

Figure S7. Analysis of the enzymatic fragments of peptide 5-t-(β-D-Glc) in the presence of Chymotrypsin by LC-MS (ESI). Among them, t indicates D-Threonine.

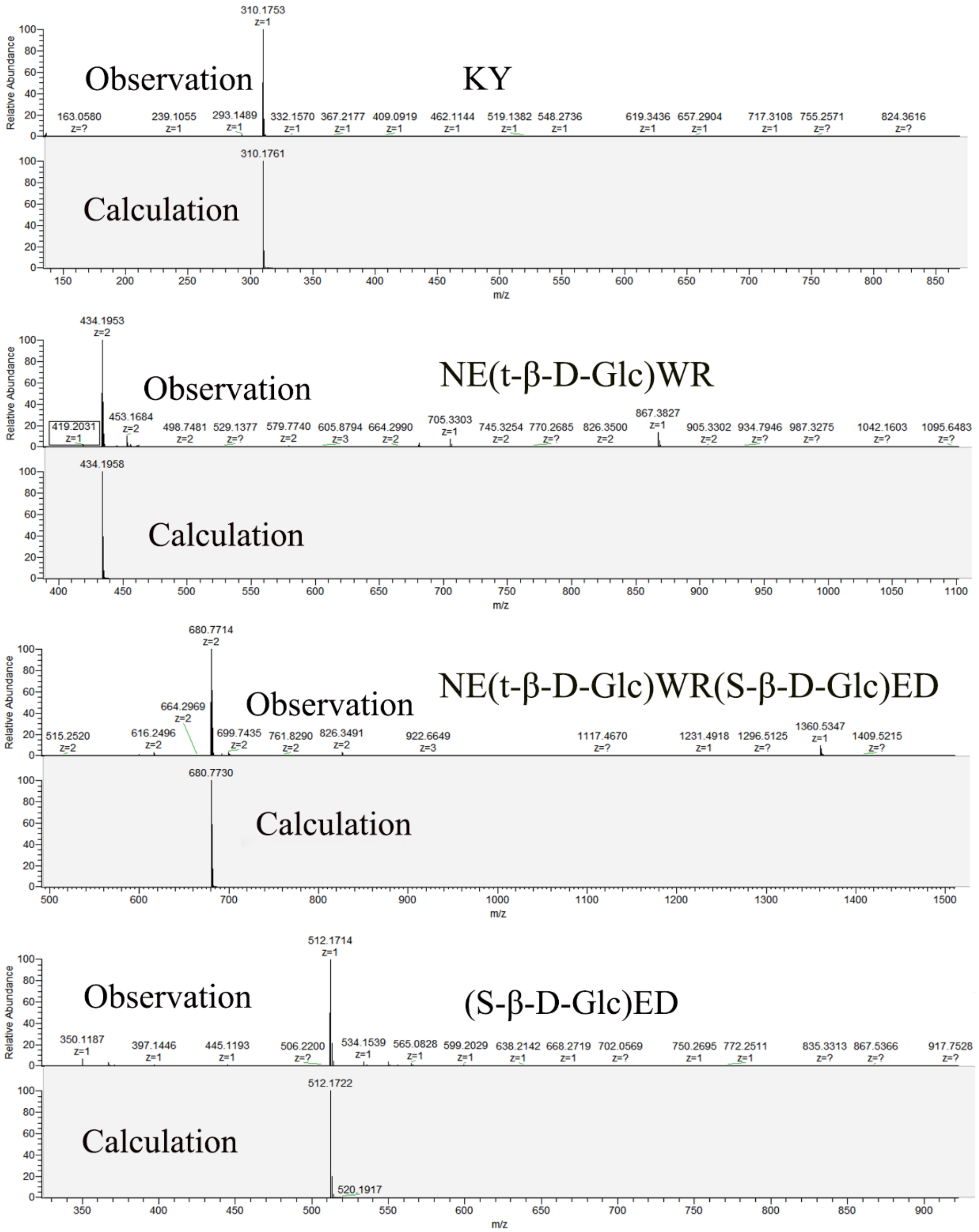

Figure S8. Analysis of the enzymatic fragments of peptide 5-t-(β-D-Glc)-(S-β-D-Glc) in the presence of Chymotrypsin by LC-MS (ESI). Among them, t indicates D-Threonine.

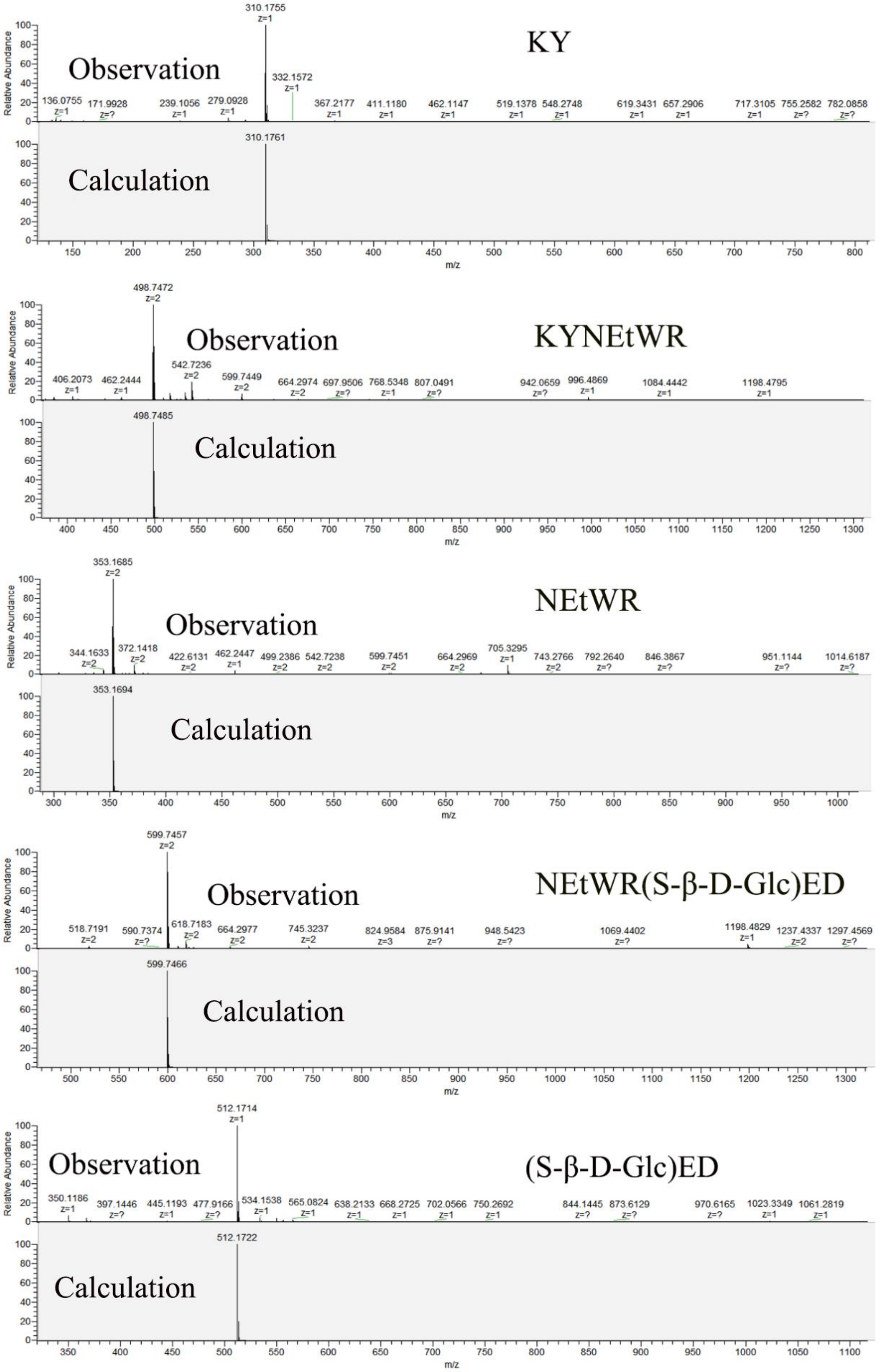

Figure S9. Analysis of the enzymatic fragments of peptide 5-t-(S-β-D-Glc) in the presence of Chymotrypsin by LC-MS (ESI). Among them, t indicates D-Threonine.

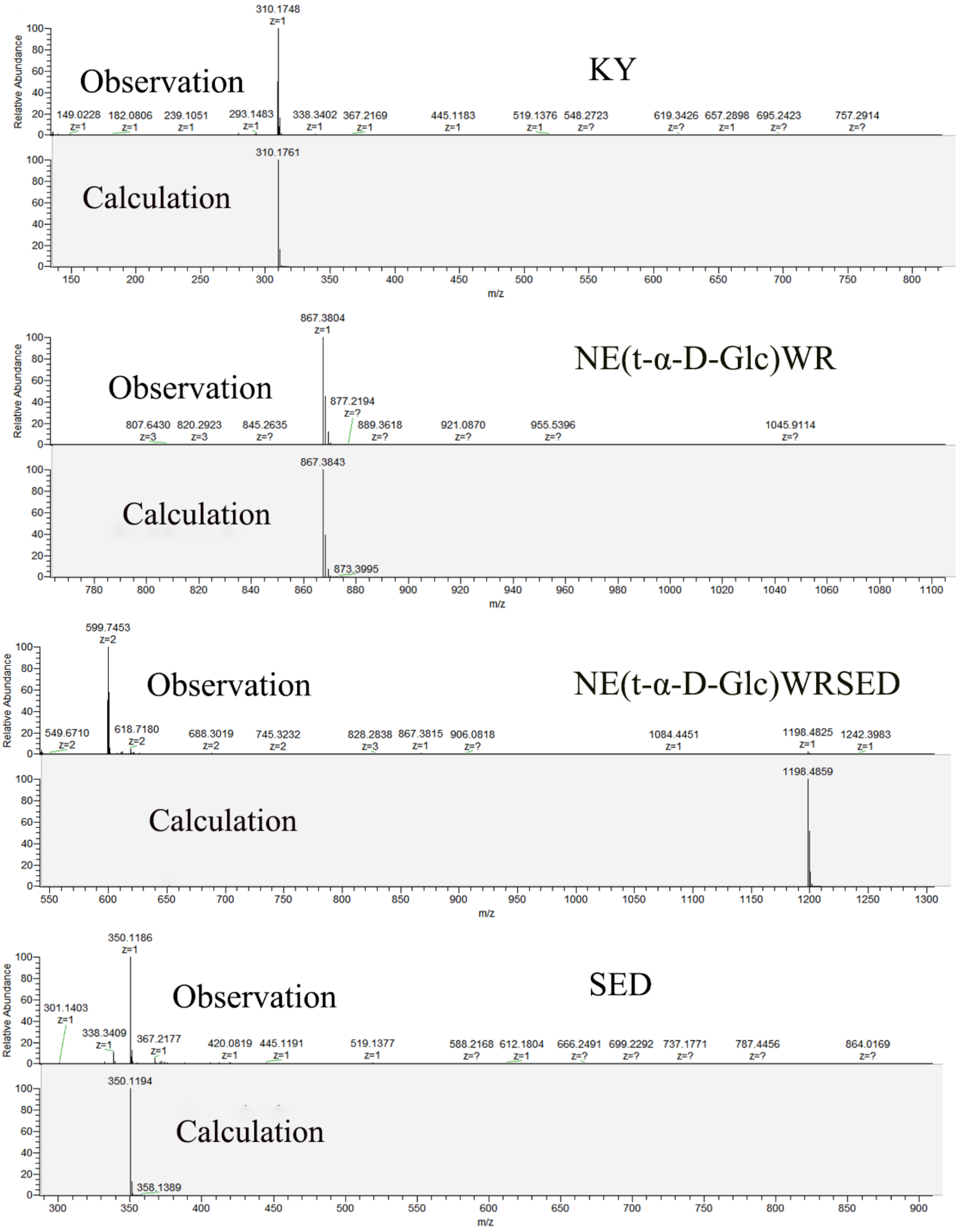

Figure S10. Analysis of the enzymatic fragments of peptide 5-t-(α-D-Glc) in the presence of Chymotrypsin by LC-MS (ESI). Among them, t indicates D-Threonine.

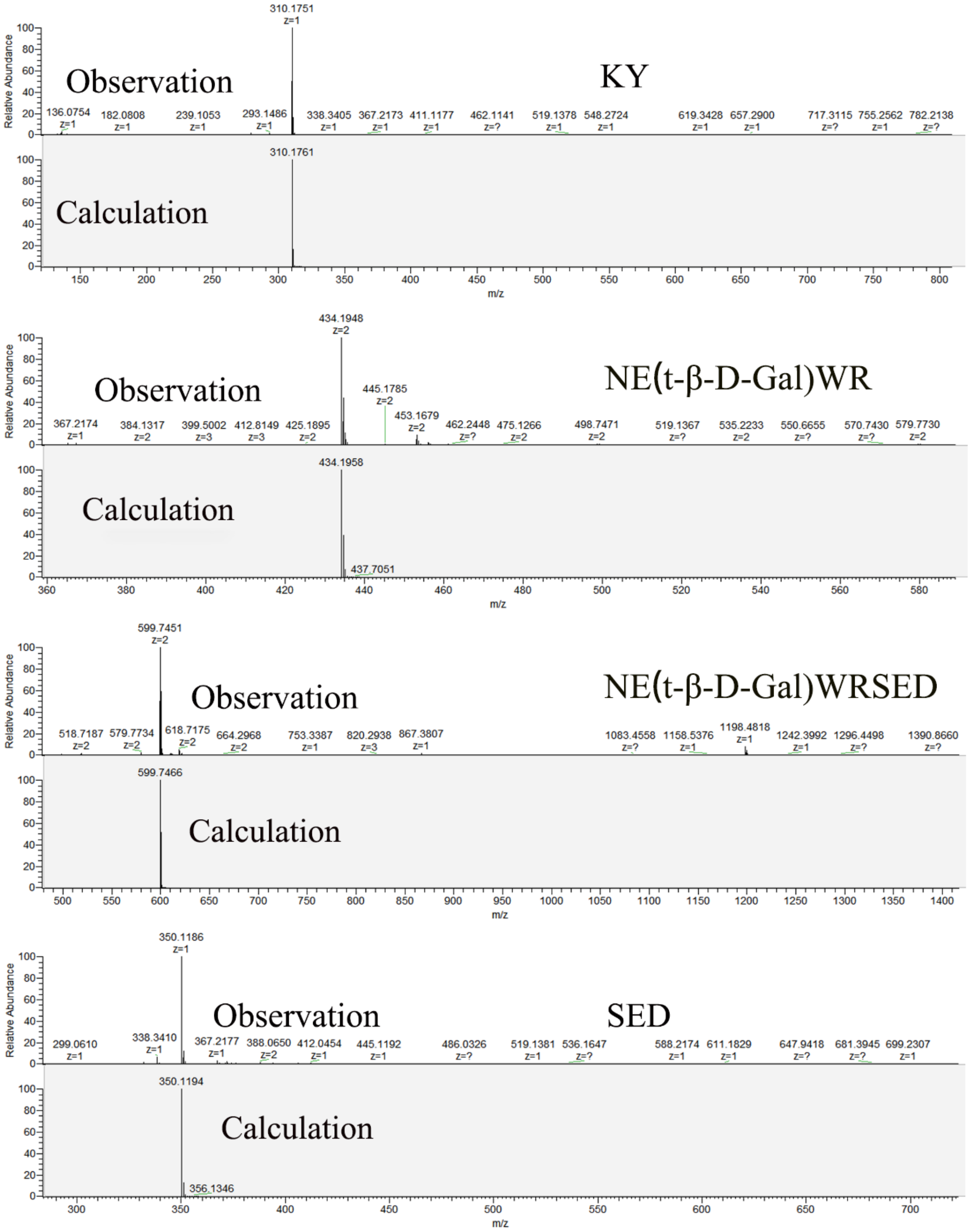

Figure S11. Analysis of the enzymatic fragments of peptide 5-t-(β-D-Gal) in the presence of Chymotrypsin by LC-MS (ESI). Among them, t indicates D-Threonine.

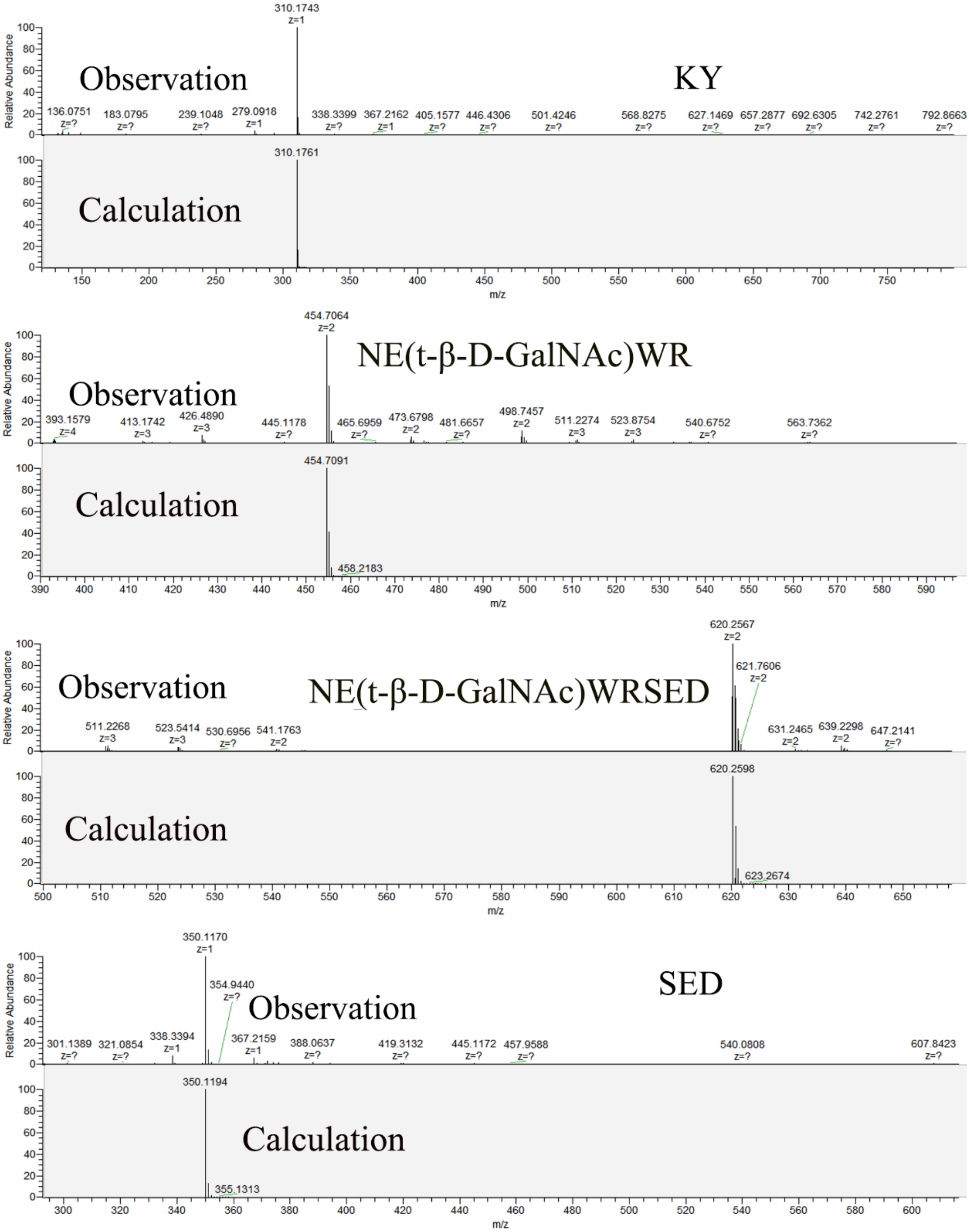

Figure S12. Analysis of the enzymatic fragments of peptide 5-t-(β-D-GalNAc) in the presence of Chymotrypsin by LC-MS (ESI). Among them, t indicates D-Threonine.

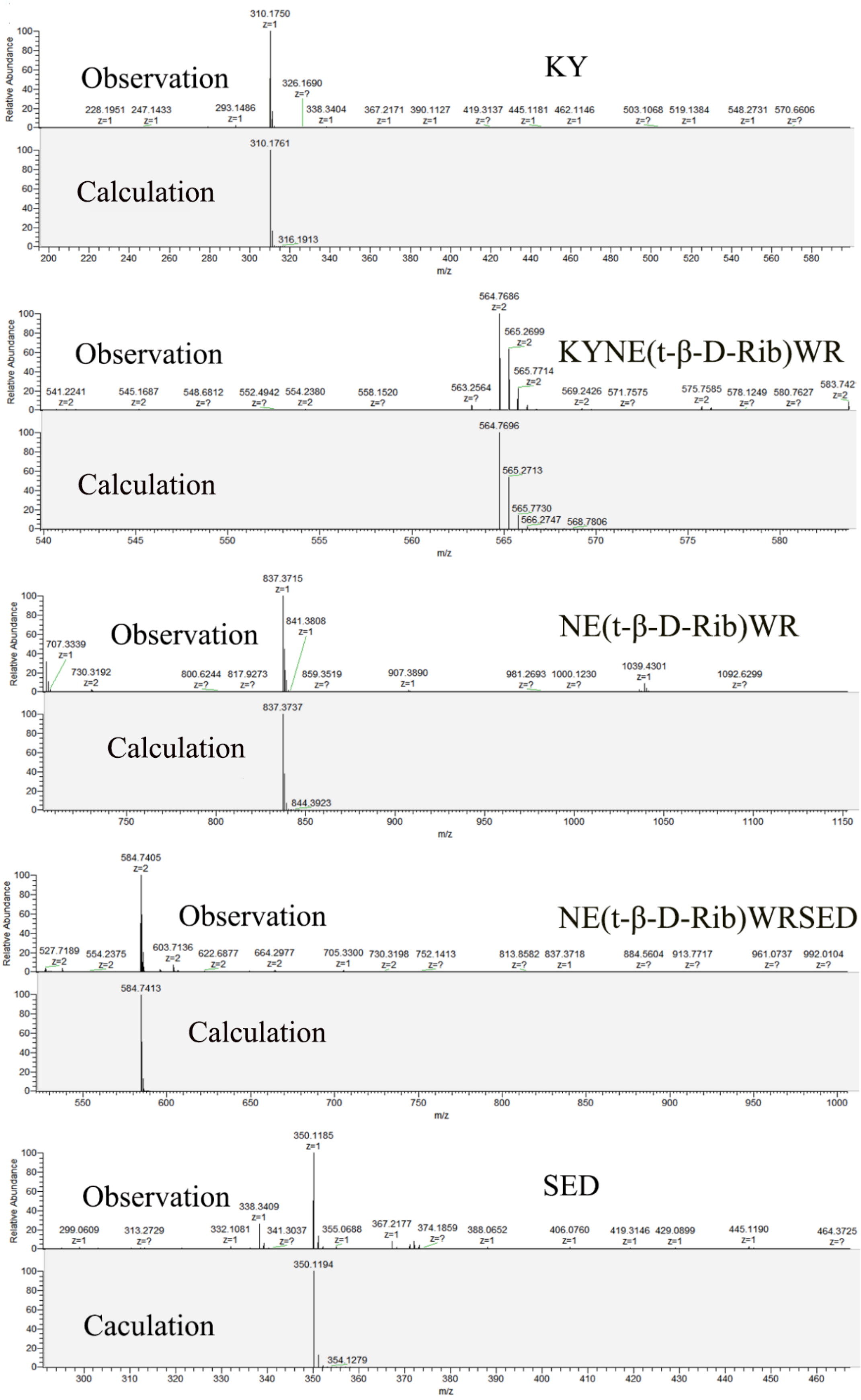

Figure S13. Analysis of the enzymatic fragments of peptide 5-t-(β-D-Rib) in the presence of Chymotrypsin by LC-MS (ESI). Among them, t indicates D-Threonine.

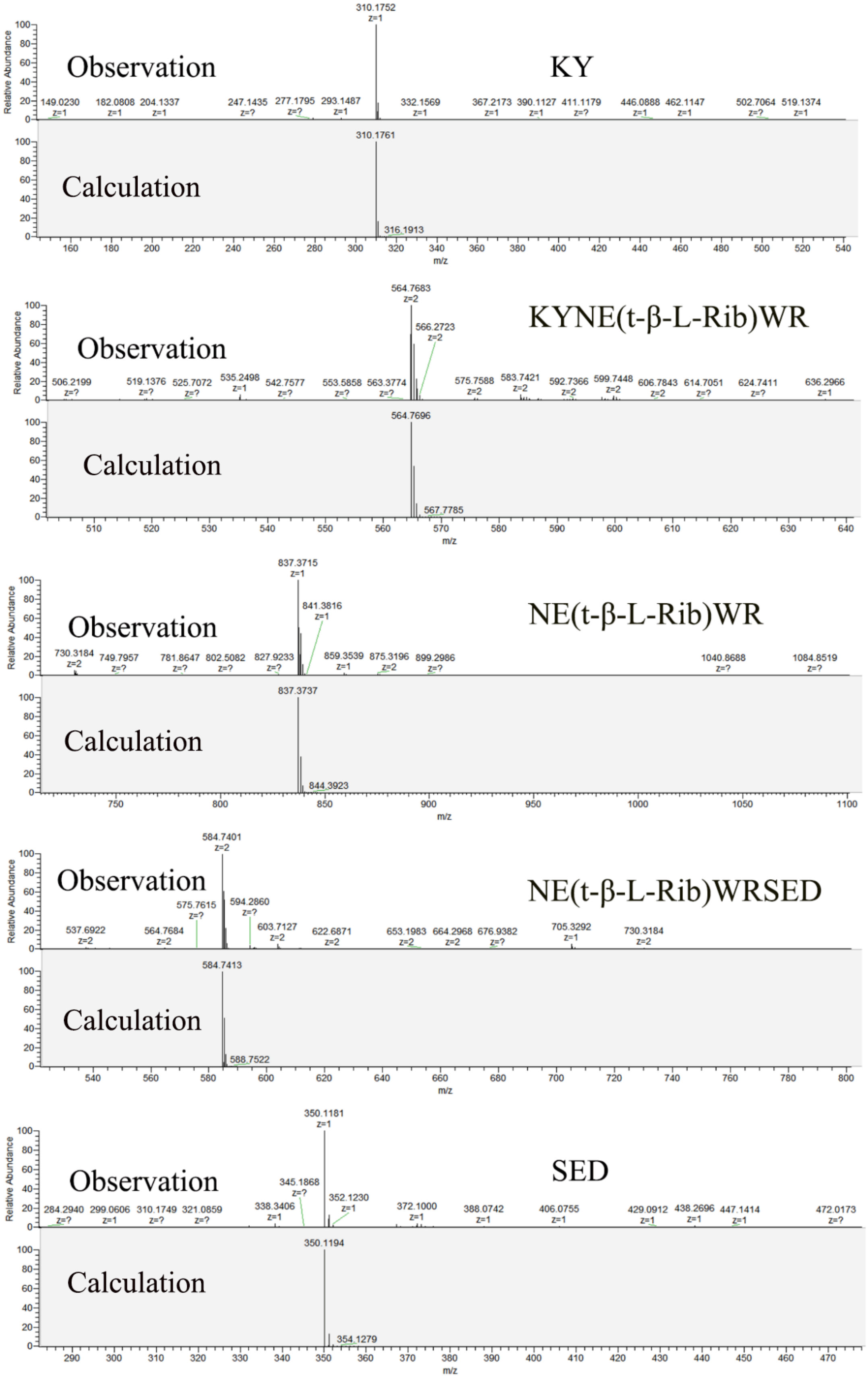

Figure S14. Analysis of the enzymatic fragments of peptide 5-t-(β-L-Rib) in the presence of Chymotrypsin by LC-MS (ESI). Among them, t indicates D-Threonine.

Table S3. Analysis of the interaction between 5-t and chymotrypsin.

Entry	Residue_Rec	Residue_Lig	Distance/A	Interaction type
1	His40	Lys1	2.25	Hydrogen bonding
2	His40	Tyr2	2.33	Hydrogen bonding
3	Tyr94	Arg7	2.29	Hydrogen bonding
4	Ser96	Arg7	2.54	Hydrogen bonding
5	Gly193	Asp10	2.22	Hydrogen bonding
6	Ser195	Asp10	2.14	Hydrogen bonding
7	Gly216	Asp10	2.40	Hydrogen bonding
8	His57	Glu9	4.76	Salt Bridge
9	Phe39	Glu4	3.46	Hydrophobic Interaction
10	Phe41	Glu4	3.49	Hydrophobic Interaction

Table S4. Analysis of the interaction between 5-t-(β-D-Glc) and chymotrypsin.

Entry	Residue_Rec	Residue_Lig	Distance/A	Interaction type
1	His57	Arg7	2.46	Hydrogen bonding
2	Thr61	Tyr2	2.07	Hydrogen bonding
3	Tyr94	Arg7	2.05	Hydrogen bonding
4	Gly193	Thr5	2.12	Hydrogen bonding
5	Asp194	Thr5	2.58	Hydrogen bonding
6	Ser195	Thr5	1.97	Hydrogen bonding
7	Ser195	Thr5	2.41	Hydrogen bonding
8	Gly216	Thr5	2.09	Hydrogen bonding
9	Gly216	Thr5	2.58	Hydrogen bonding
10	Lys90	Asp10	4.45	Salt Bridges
11	Asp35	Tyr2	3.43	Hydrophobic Interaction
12	Ile99	Trp6	3.37	Hydrophobic Interaction
13	Trp215	Trp6	4.32	π-Stacking
14	Trp215	Trp6	4.13	π-Stacking

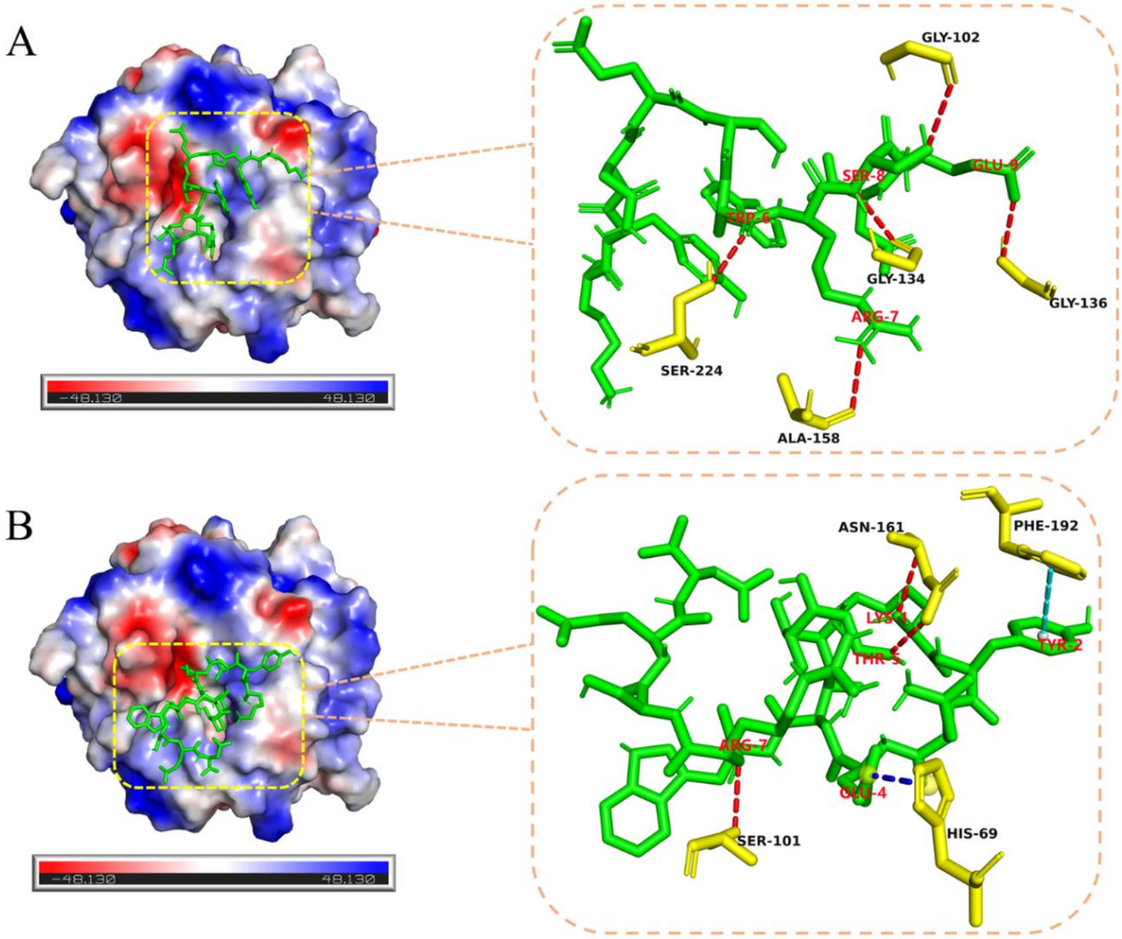

Figure S15. Computational simulation of the interaction of 5-t (A) and 5-t-(β-D-Glc) (B) with PROK, respectively. The green segment is the ligand and the yellow segment is the residue in the receptor. The red characters in the figure belong to the ligand amino acids and the black characters belong to the receptor amino acids. Hydrogen bonds (red, ---), salt bridges (blue, ---), and π-π stacking (blue-green, ---).

Mass spectrometry results of the glycopeptides synthesized in the experiment as well as the glycosylated amino acids.

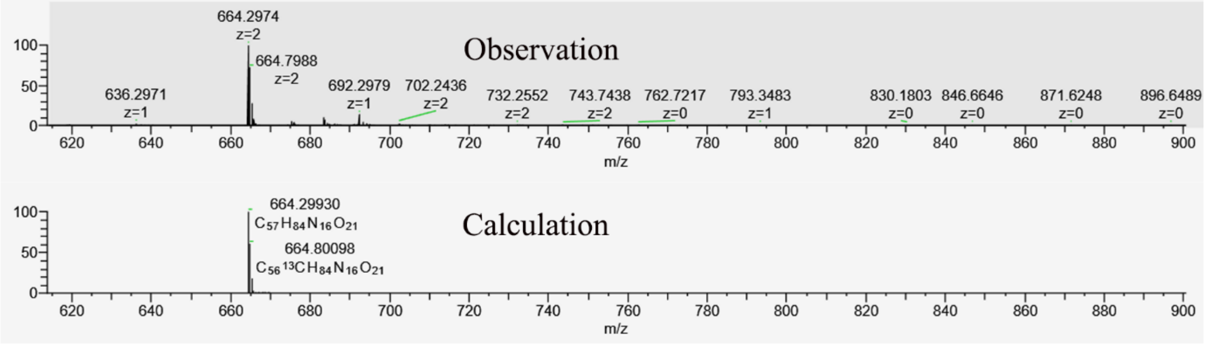

Figure S16. ESI-MS data of peptide all-L.

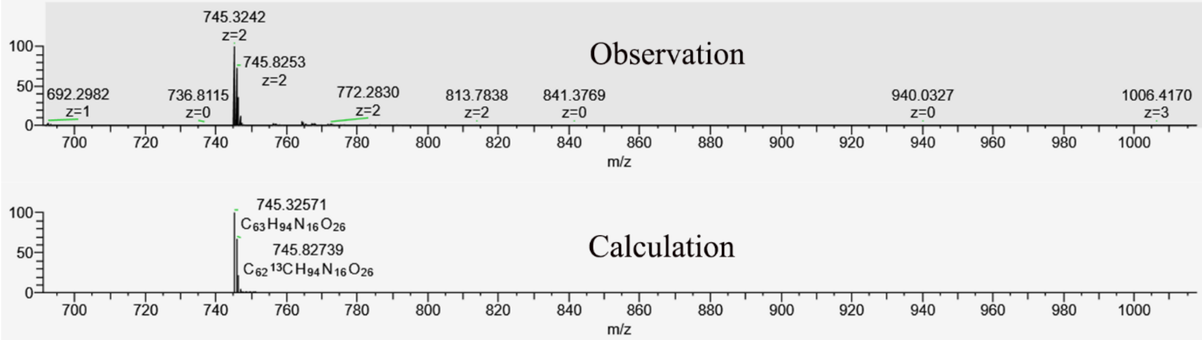

Figure S17. ESI-MS data of peptide all-L-(T-β-D-Glc).

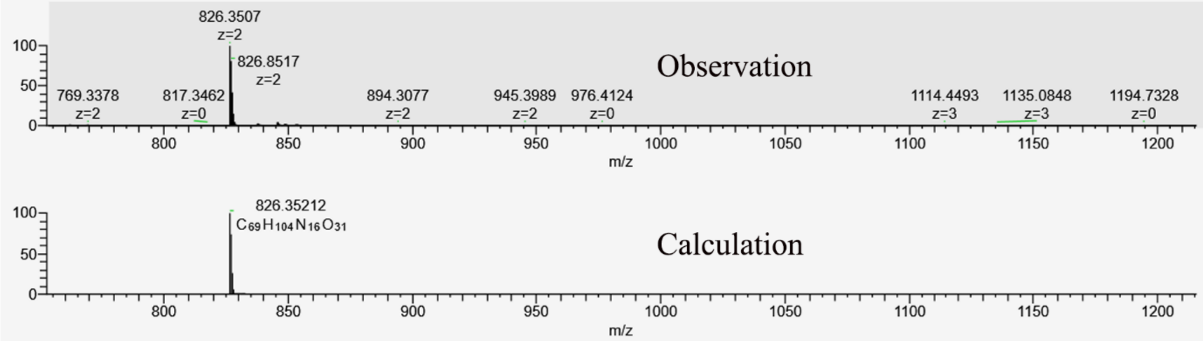

Figure S18. ESI-MS data of peptide all-L-(T-β-D-Glc)-(S-β-D-Glc).

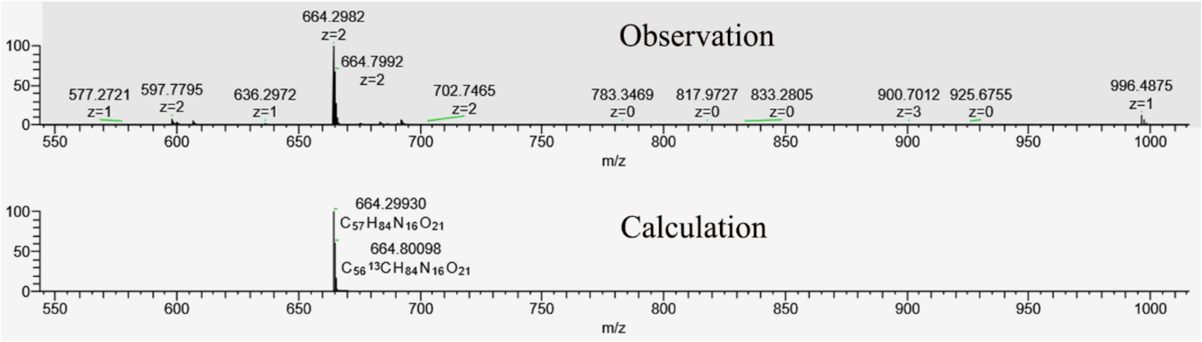

Figure S19. ESI-MS data of peptide 5-t.

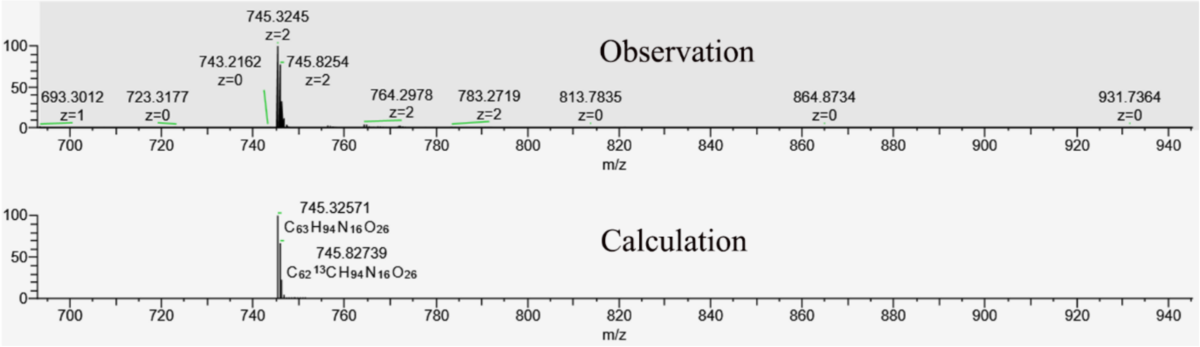

Figure S20. ESI-MS data of peptide 5-t-(β-D-Glc).

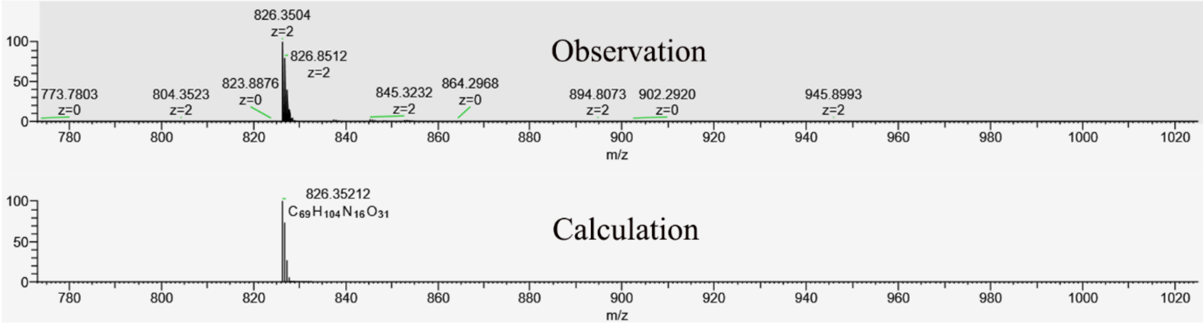

Figure S21. ESI-MS data of peptide 5-t-(β-D-Glc)-(S-β-D-Glc).

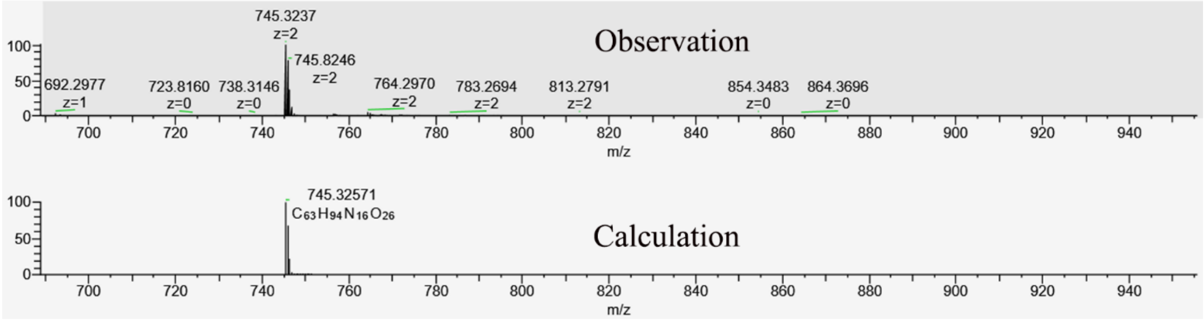

Figure S22. ESI-MS data of peptide 5-t-(S-β-D-Glc).

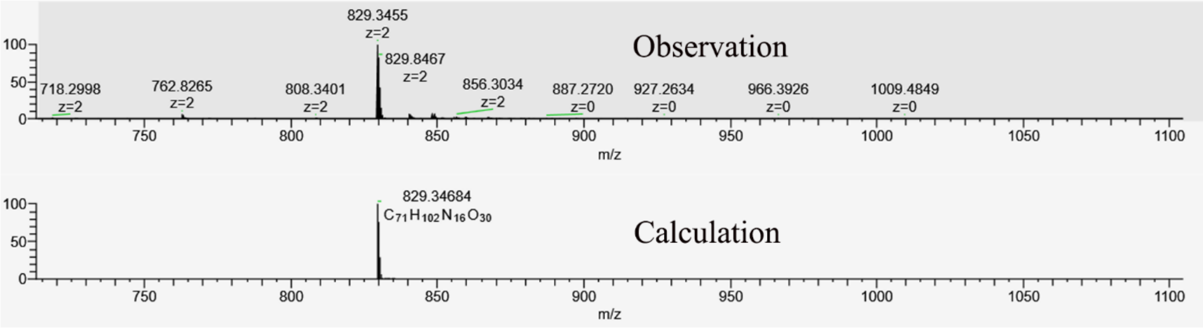

Figure S23. ESI-MS data of peptide 5-t-(β-D-Glc-Ac4).

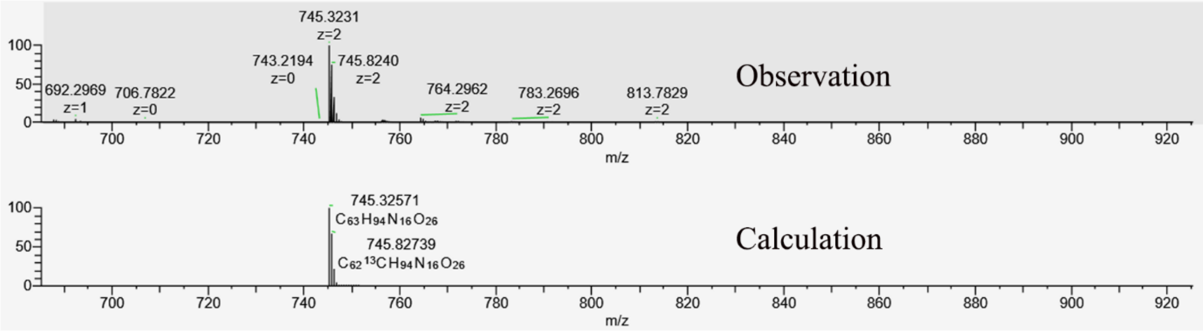

Figure S24. ESI-MS data of peptide 5-t-(α-D-Glc).

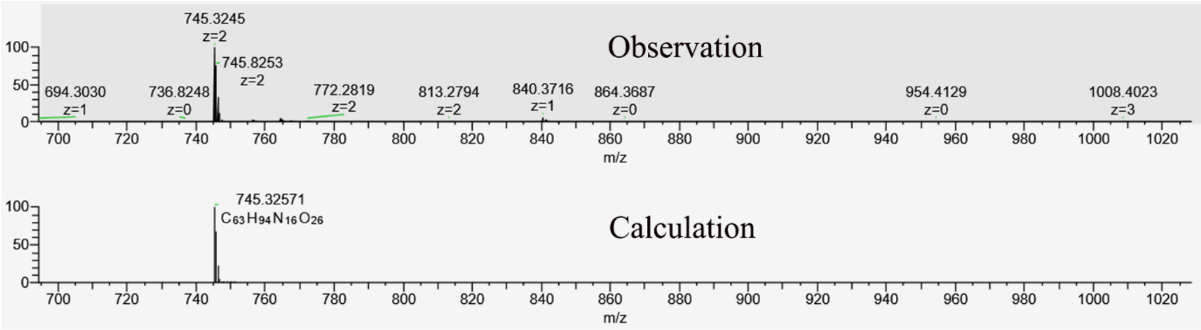

Figure S25. ESI-MS data of peptide 5-t-(β-D-Gal).

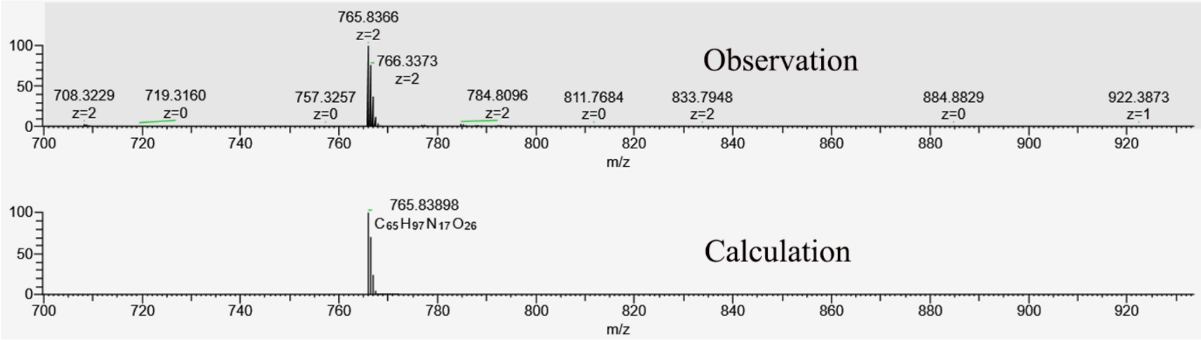

Figure S26. ESI-MS data of peptide 5-t-(β-D-GalNAc).

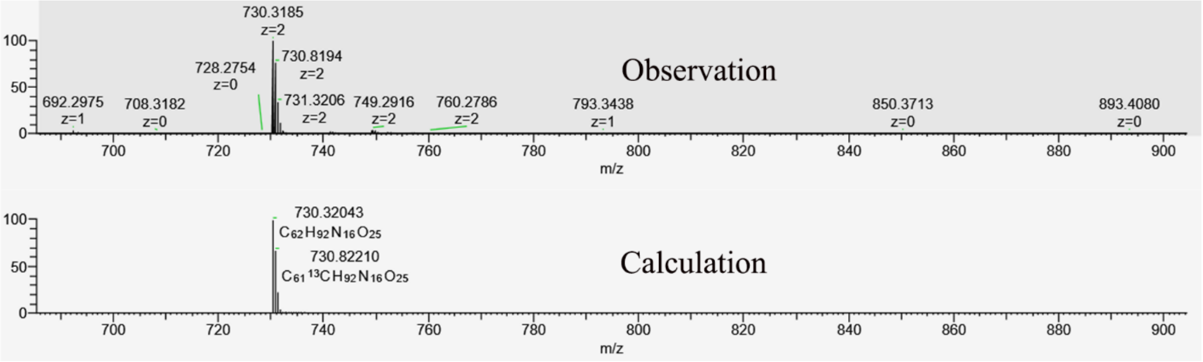

Figure S27. ESI-MS data of peptide 5-t-(β-D-Rib).

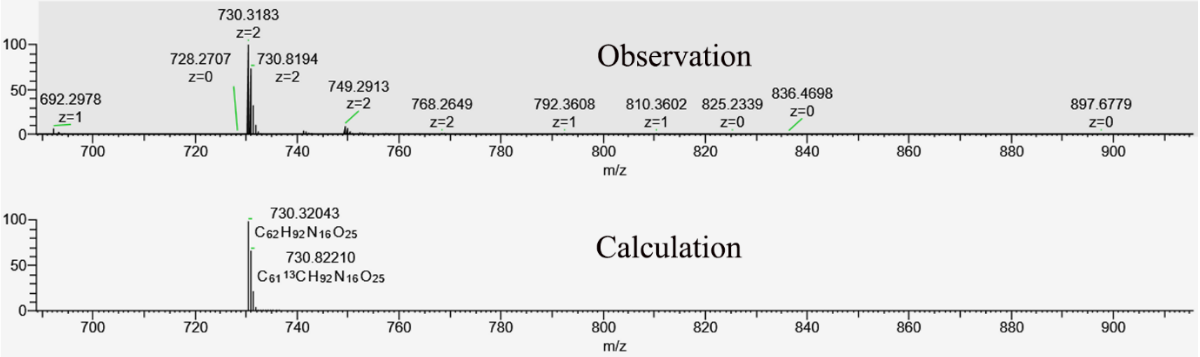

Figure S28. ESI-MS data of peptide 5-t-(β-L-Rib).

^{1}H-NMR, ^{13}C-NMR, ESI-MS for glycoamino acids

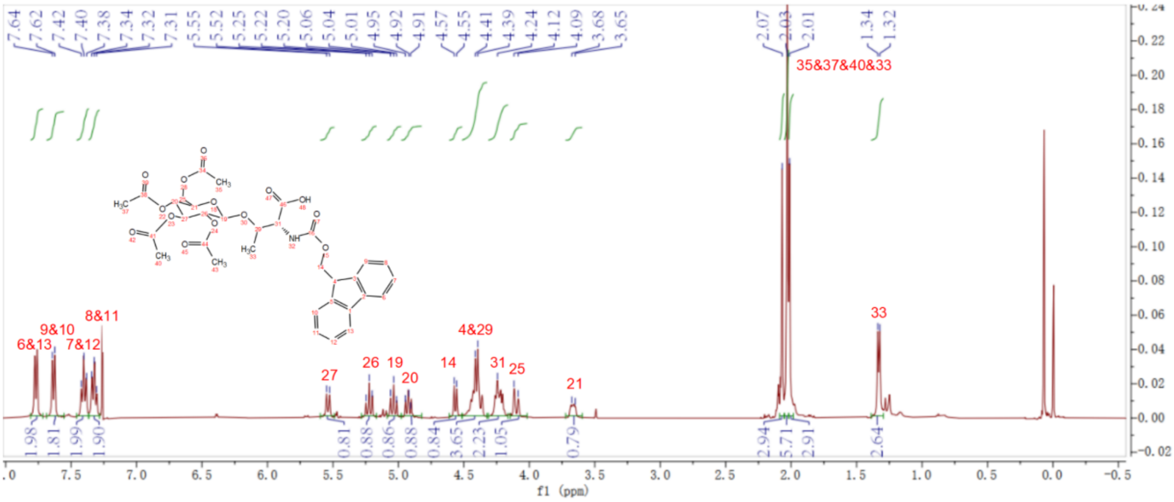

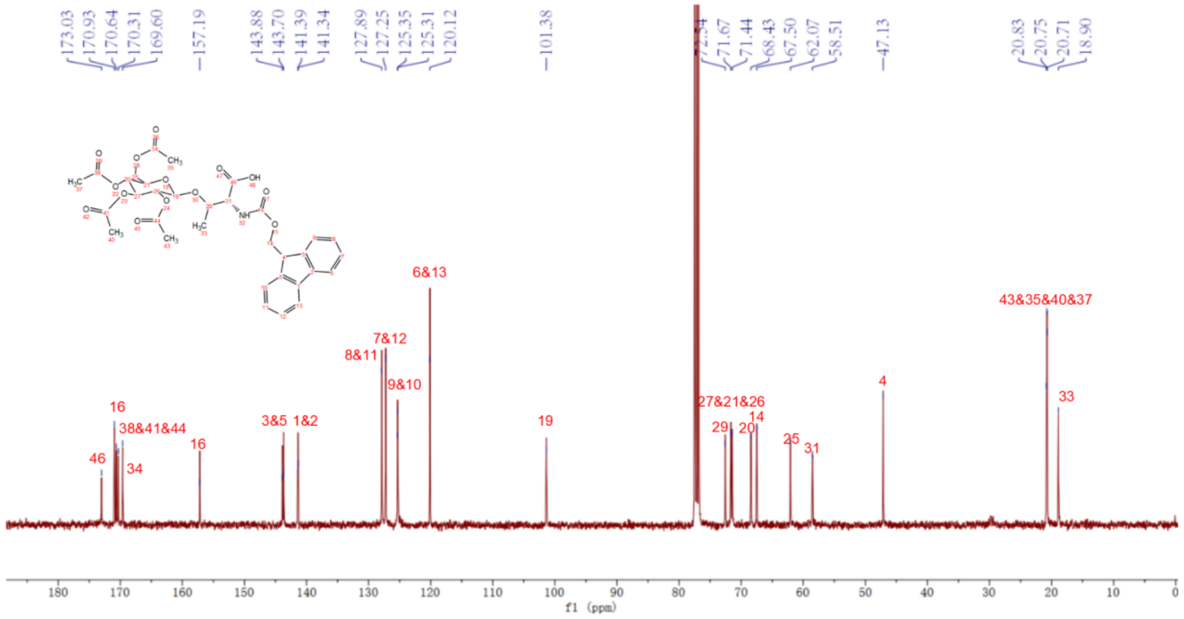

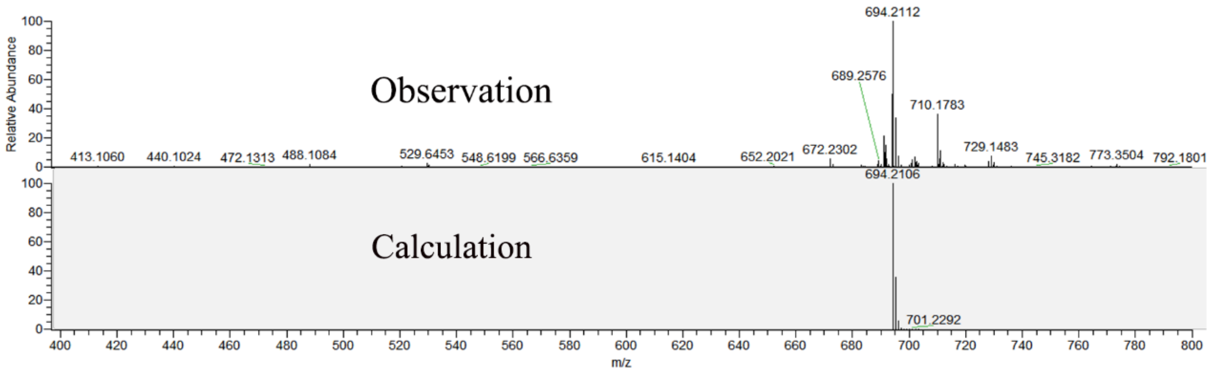

Figure S29. ^{1}H-NMR, ^{13}C-NMR, ESI-MS of **Fmoc-D-Thr-[β-D-Glc(Ac$_4$)]-OH**. (^{1}H NMR (400 MHz, Chloroform-*d*)-- δ 7.77 (d, *J* = 7.4 Hz, 2H), 7.63 (d, *J* = 7.3 Hz, 2H), 7.40 (t, *J* = 7.3 Hz, 2H), 7.32 (t, *J* = 7.0 Hz, 2H), 5.54 (d, *J* = 9.5 Hz, 1H), 5.22 (t, *J* = 9.6 Hz, 1H), 5.04 (t, *J* = 9.8 Hz, 1H), 4.92 (t, *J* = 8.7 Hz, 1H), 4.56 (d, *J* = 8.0 Hz, 1H), 4.51 – 4.32 (m, 4H), 4.31 – 4.16 (m, 2H), 4.15 – 4.02 (m, 1H), 3.72 – 3.60 (m, 1H), 2.07 (s, 3H), 2.03 (s, 6H), 2.01 (s, 3H), 1.33 (d, *J* = 6.2 Hz, 3H); ^{13}C NMR (100 MHz, Chloroform-*d*)-- δ 173.03, 170.93, 170.64, 170.31, 169.60, 157.19, 143.88, 143.70, 141.39, 141.34, 127.89, 127.25, 125.35, 125.31, 120.12, 101.38, 72.54, 71.67, 71.44, 68.43, 67.50, 62.07, 58.51, 47.13, 20.83, 20.75, 20.71, 18.90). HRMS (ESI): m/z ([M+Na]$^{+}$) calculated for $C_{33}H_{37}NO_{14}$: 694.2106; found: 694.2112.

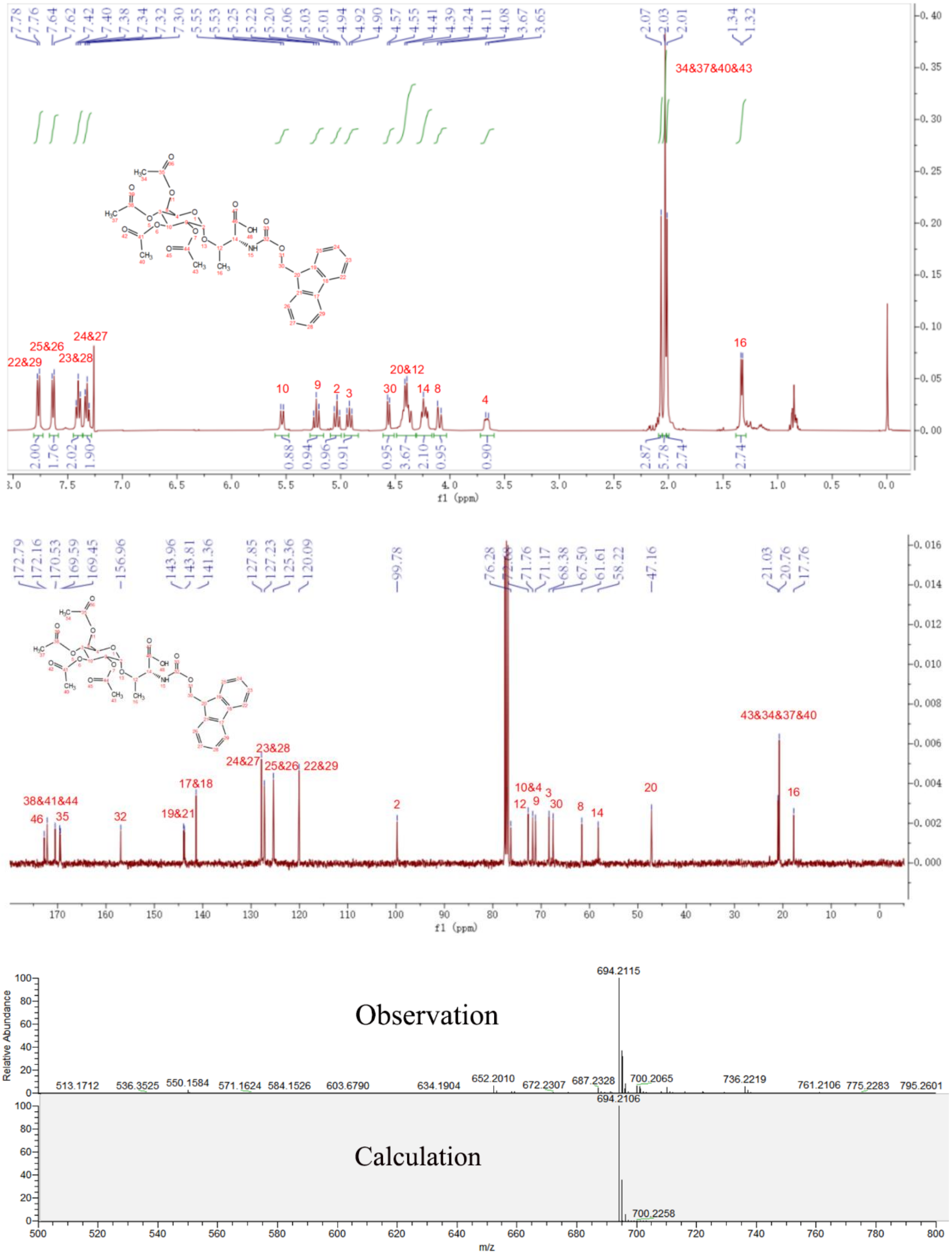

Figure S30. ^{1}H-NMR, ^{13}C-NMR, ESI-MS of **Fmoc-D-Thr-[α-D-Glc(Ac4)]-OH**. (^{1}H NMR (400 MHz, Chloroform-*d*)-- δ 7.77 (d, *J* = 7.4 Hz, 2H), 7.63 (d, *J* = 7.4 Hz, 2H), 7.40 (t, *J* = 7.4 Hz, 2H), 7.32 (t, *J* = 7.3 Hz, 2H), 5.54 (d, *J* = 9.3 Hz, 1H), 5.22 (t, *J* = 9.6 Hz, 1H), 5.03 (t, *J* = 9.7 Hz, 1H), 4.92 (t, *J* = 8.9 Hz, 1H), 4.56 (d, *J* = 7.8 Hz, 1H), 4.49 – 4.31 (m, 4H), 4.30 – 4.02 (m, 3H), 3.72 – 3.57 (m, 1H), 2.07 (s, 3H), 2.03 (s, 6H), 2.01 (s, 3H), 1.33 (d, *J* = 6.4 Hz,

3H); ^{13}C NMR (100 MHz, Chloroform-*d*)-- δ 172.79, 172.16, 170.53, 169.59, 169.45, 156.96, 143.96, 143.81, 141.36, 127.85, 127.23, 125.36, 120.09, 99.78, 76.28, 72.68, 71.76, 71.17, 68.38, 67.50, 61.61, 58.22, 47.16, 21.03, 20.76, 17.76). HRMS (ESI): m/z ([M+Na]$^+$) calculated for $C_{33}H_{37}NO_{14}$: 694.2106; found: 694.2115.

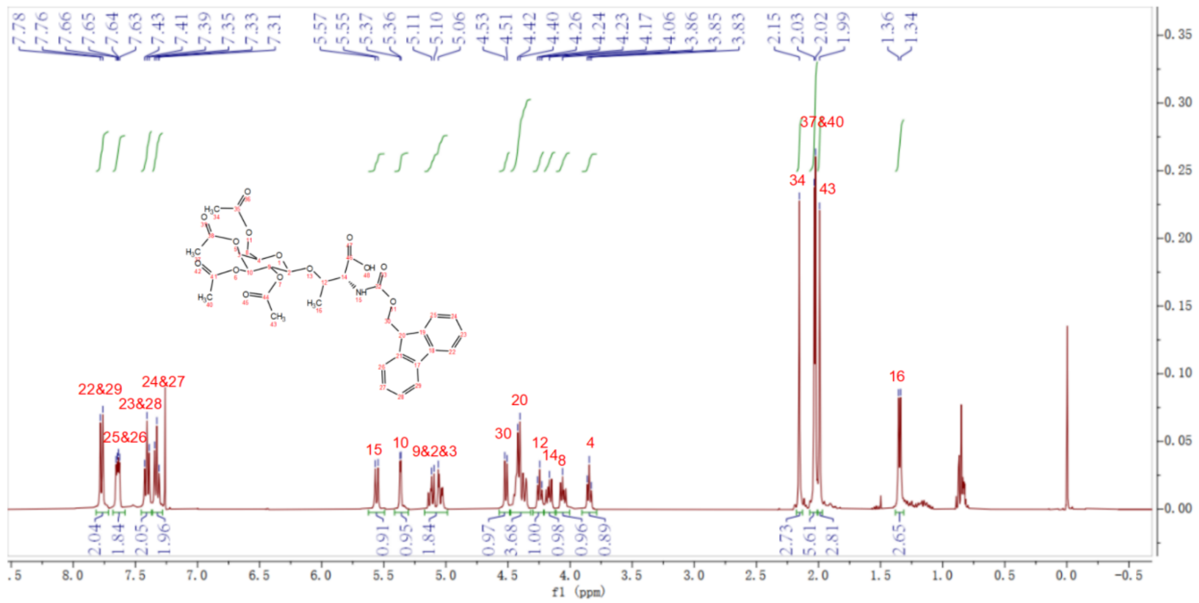

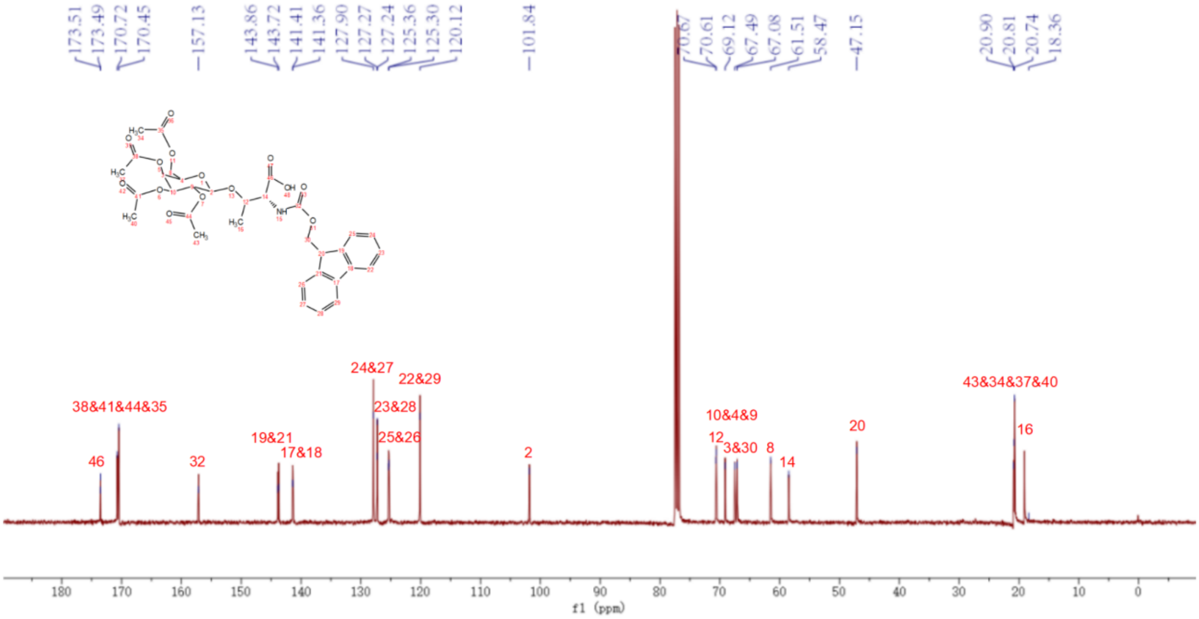

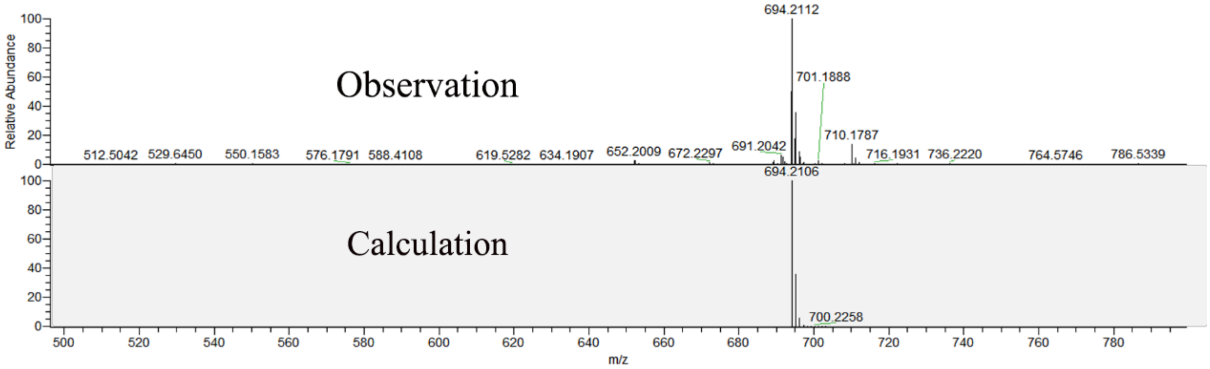

Figure S31. ^{1}H-NMR, ^{13}C-NMR, ESI-MS of **Fmoc-D-Thr-[β-D-Gal(Ac$_4$)]-OH.**(^{1}H NMR (400 MHz, Chloroform-*d*)-- δ 7.77 (d, *J* = 7.5 Hz, 2H), 7.68 – 7.58 (m, 2H), 7.41 (t, *J* = 7.4 Hz, 2H), 7.33 (t, *J* = 7.3 Hz, 2H), 5.56 (d, *J* = 9.4 Hz, 1H), 5.37 (d, *J* = 3.1 Hz, 1H), 5.17 – 4.99 (m, 2H), 4.52 (d, *J* = 7.7 Hz, 1H), 4.48 – 4.32 (m, 4H), 4.28 – 4.02 (m, 3H), 3.85 (t, *J* = 6.7 Hz, 1H), 2.15 (s, 3H), 2.03 (d, *J* = 4.0 Hz, 6H), 1.99 (s, 3H), 1.35 (d, *J* = 6.4 Hz, 3H); ^{13}C NMR (100 MHz, Chloroform-*d*)-- δ 173.51, 173.49, 170.72, 170.45, 157.13, 143.86, 143.72, 141.41, 141.36, 127.90, 127.27, 127.24, 125.36, 125.30, 120.12, 101.84, 70.67, 70.61, 69.12, 67.49, 67.08, 61.51, 58.47, 47.15, 20.90, 20.81, 20.74, 18.36). HRMS (ESI): m/z ([M+Na]$^{+}$) calculated for $C_{33}H_{37}NO_{14}$: 694.2106; found: 694.2112.

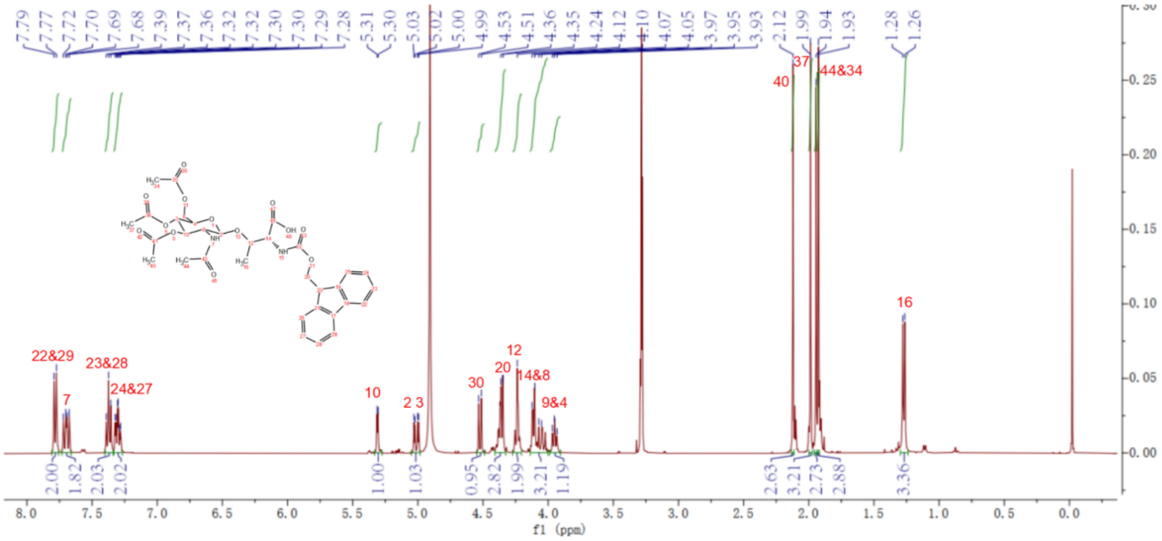

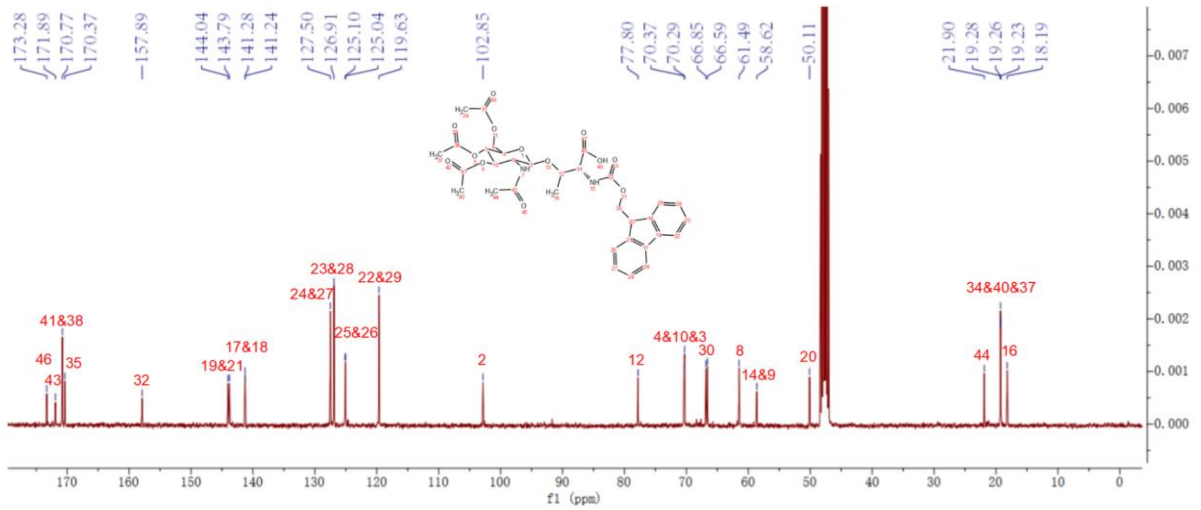

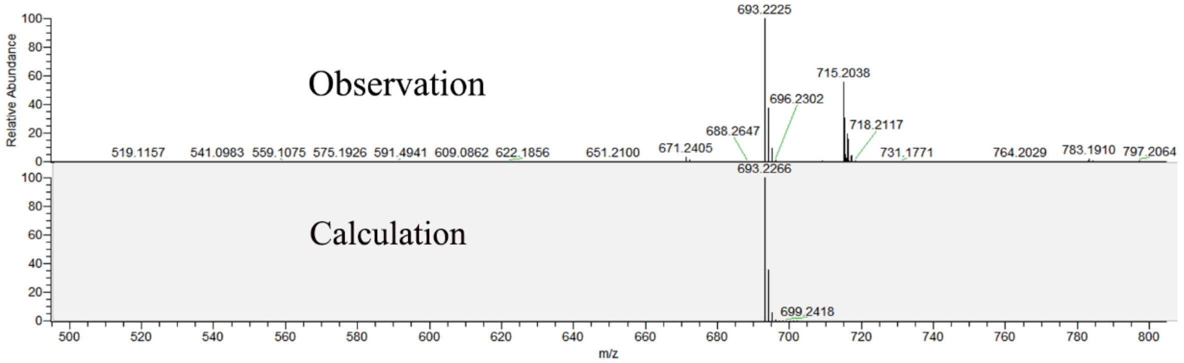

Figure S32. ^{1}H-NMR, ^{13}C-NMR, ESI-MS of **Fmoc-D-Thr-[β-D-Gal(Ac$_3$)NAc]-OH**. (^{1}H NMR (400 MHz, Methanol-*d*$_4$)-- δ 7.78 (d, *J* = 7.5 Hz, 2H), 7.73 – 7.66 (m, 2H), 7.37 (t, *J* = 7.1 Hz, 2H), 7.30 (t, *J* = 7.4, 3.0 Hz, 2H), 5.31 (d, *J* = 3.4 Hz, 1H), 5.01 (dd, *J* = 11.3, 3.3 Hz, 1H), 4.52 (d, *J* = 8.4 Hz, 1H), 4.41 – 4.33 (m, 3H), 4.27 – 4.20 (m, 2H), 4.14 – 4.00 (m, 3H), 3.99 – 3.91 (m, 1H), 2.12 (s, 3H), 1.99 (s, 3H), 1.94 (s, 3H), 1.93 (s, 3H), 1.27 (d, *J* = 6.5 Hz, 3H). ^{13}C NMR (100 MHz, Methanol-*d*$_4$) -- δ 173.28, 171.89, 170.77, 170.37, 157.89, 144.04, 143.79, 141.28, 141.24, 127.50, 126.91, 125.10, 125.04, 119.63, 102.85, 77.80, 70.37, 70.29, 66.85, 66.59, 61.49, 58.62, 50.11, 21.90, 19.28, 19.26, 19.23, 18.19. HRMS (ESI): m/z ([M+Na]$^+$) calculated for $C_{33}H_{38}N_2O_{13}$: 693.2266; found: 693.2225.

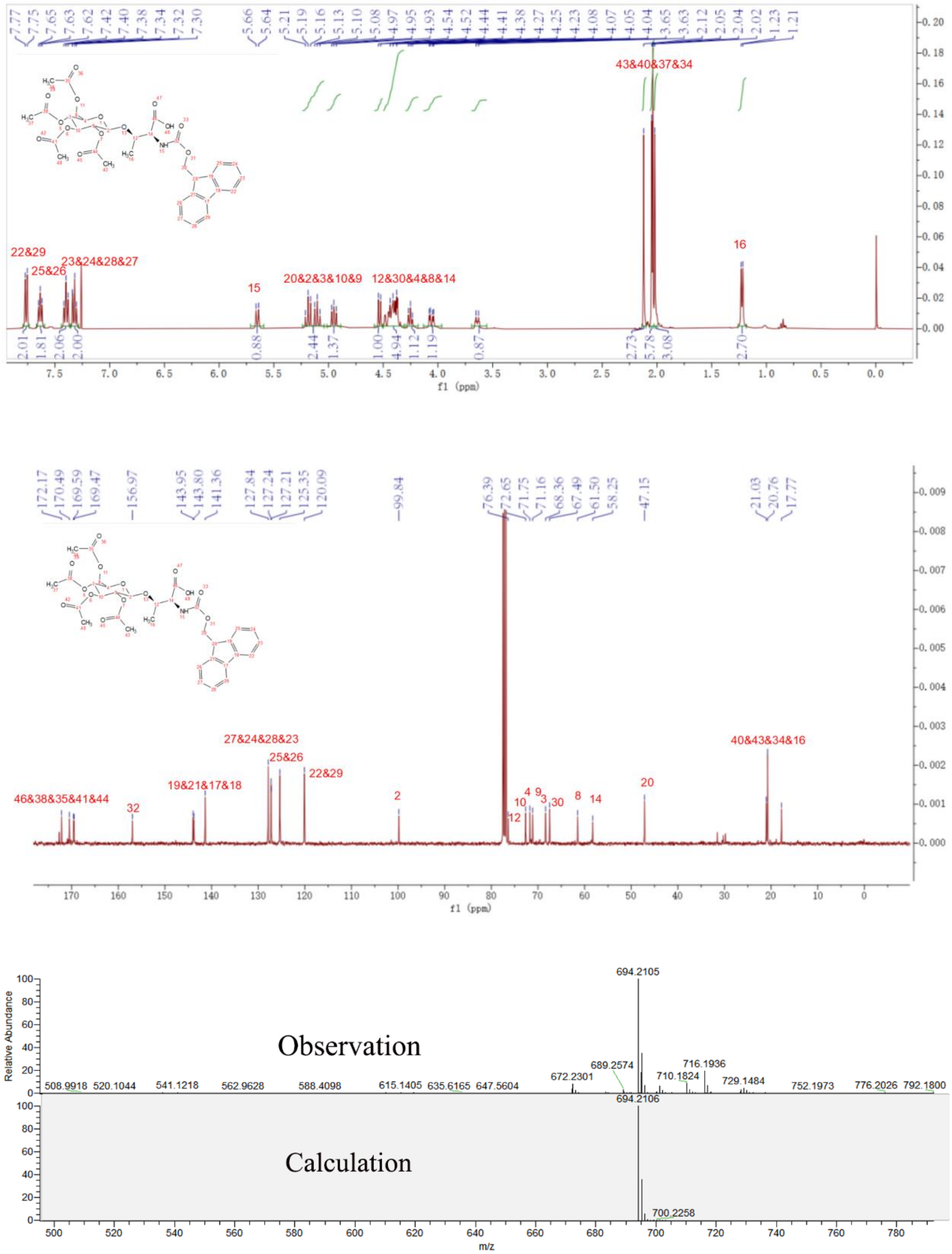

Figure S33. ^{1}H-NMR, ^{13}C-NMR, ESI-MS of **Fmoc-$_{\text{L}}$-Thr-[β-D-Glc(Ac$_4$)]-OH**. (^{1}H NMR (400 MHz, Chloroform-*d*)-- δ 7.76 (d, *J* = 7.5 Hz, 2H), 7.63 (t, *J* = 6.6 Hz, 2H), 7.40 (t, *J* = 7.5 Hz, 2H), 7.32 (t, *J* = 7.0 Hz, 2H), 5.65 (d, *J* = 9.4 Hz, 1H), 5.24 – 5.04 (m, 2H), 4.95 (t, *J* = 8.5 Hz, 1H), 4.53 (d, *J* = 8.0 Hz, 1H), 4.50 – 4.31 (m, 5H), 4.25 (t, *J* = 7.4 Hz, 1H), 4.06 (dd, *J* = 12.4 Hz, 1H), 3.69 – 3.55 (m, 1H), 2.12 (s, 3H), 2.04 (d, *J* = 4.1 Hz, 6H), 2.02 (s, 3H), 1.22 (d, *J* = 6.5 Hz, 3H); ^{13}C NMR (100 MHz, Chloroform-*d*)-- δ 172.17, 170.49, 169.59, 169.47,

156.97, 143.95, 143.80, 141.36, 127.84, 127.24, 127.21, 125.35, 120.09, 99.84, 76.39, 72.65, 71.75, 71.16, 68.36, 67.49, 61.50, 58.25, 47.15, 21.03, 20.76, 17.77. HRMS (ESI): m/z ($[M+Na]^+$) calculated for $C_{33}H_{37}NO_{14}$: 694.2106; found: 694.2105.

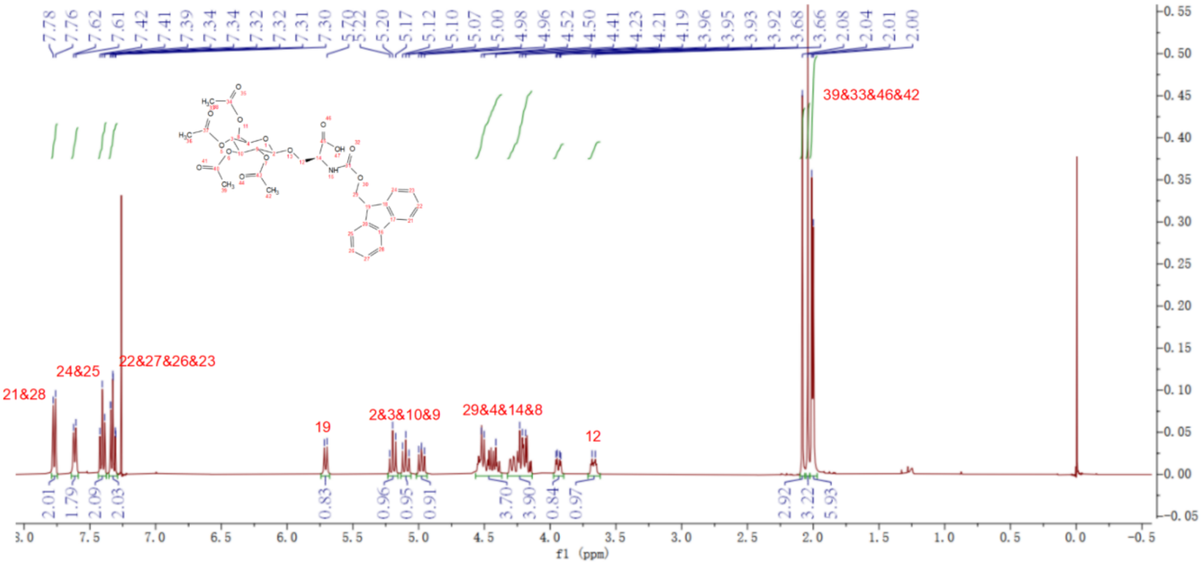

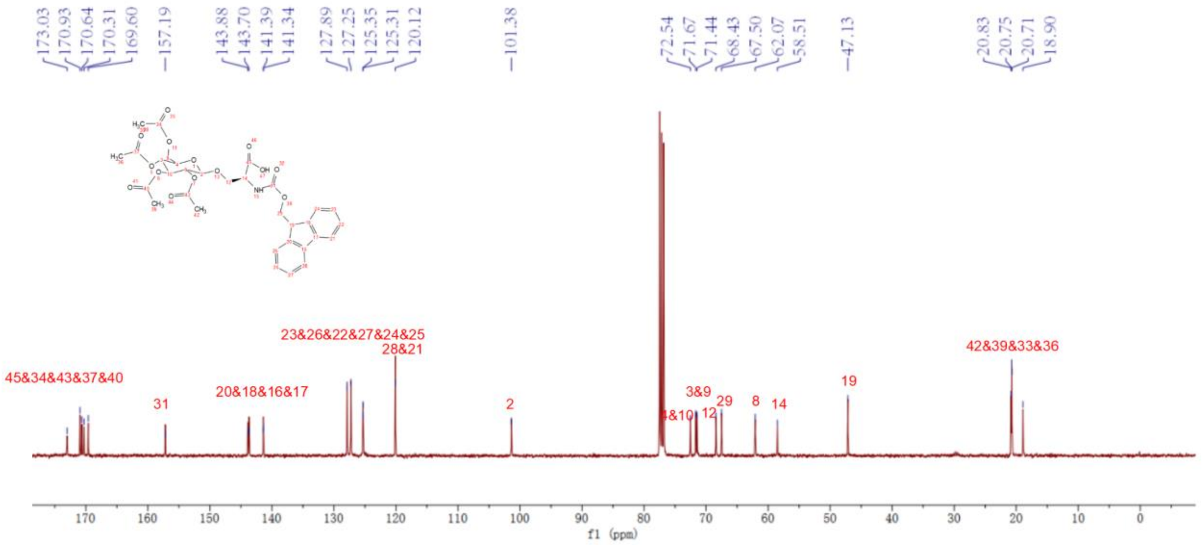

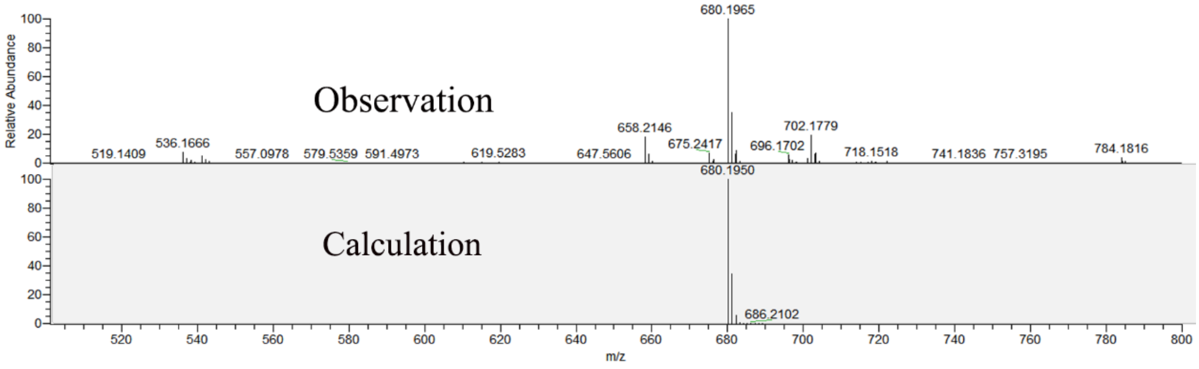

Figure S34. ^{1}H-NMR, ^{13}C-NMR, ESI-MS of **Fmoc-L-Ser-[β-D-Glc(Ac$_4$)]-OH**. (^{1}H NMR (400 MHz, Chloroform-*d*)-- δ 7.77 (d, *J* = 7.6 Hz, 2H), 7.61 (d, *J* = 7.4 Hz, 2H), 7.41 (t, *J* = 7.3 Hz, 2H), 7.32 (t, *J* = 7.3 Hz, 2H), 5.71 (d, *J* = 8.2 Hz, 1H), 5.20 (t, *J* = 9.4 Hz, 1H), 5.10 (t, *J* = 9.6 Hz, 1H), 5.03 – 4.92 (m, 1H), 4.57 – 4.37 (m, 4H), 4.33 – 4.14 (m, 4H), 3.94 (dd, *J* = 10.7, 3.7 Hz, 1H), 3.71 – 3.62 (m, 1H), 2.08 (s, 3H), 2.04 (s, 3H), 2.00 (d, *J* = 4.7 Hz, 6H); ^{13}C NMR (100 MHz, Chloroform-*d*)-- δ 173.03, 170.93, 170.64, 170.31, 169.60, 157.19, 143.88, 143.70, 141.39, 141.34, 127.89, 127.25, 125.35, 125.31, 120.12, 101.38, 72.54, 71.67, 71.44, 68.43, 67.50, 62.07, 58.51, 47.13, 20.83, 20.75, 20.71, 18.90). HRMS (ESI): m/z ([M+Na]$^{+}$) calculated for $C_{32}H_{35}NO_{14}$: 680.1950; found: 680.1965.

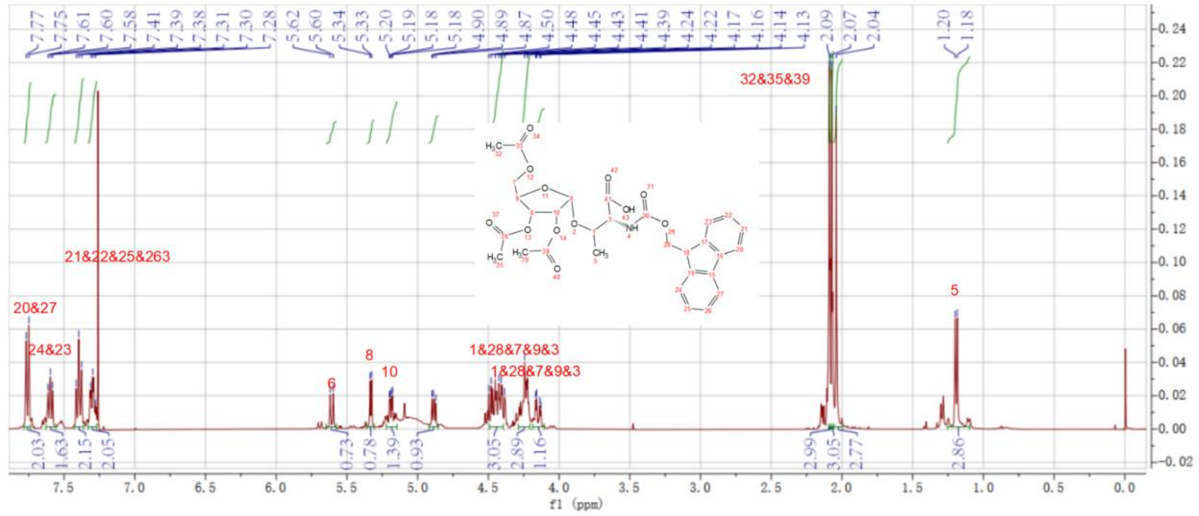

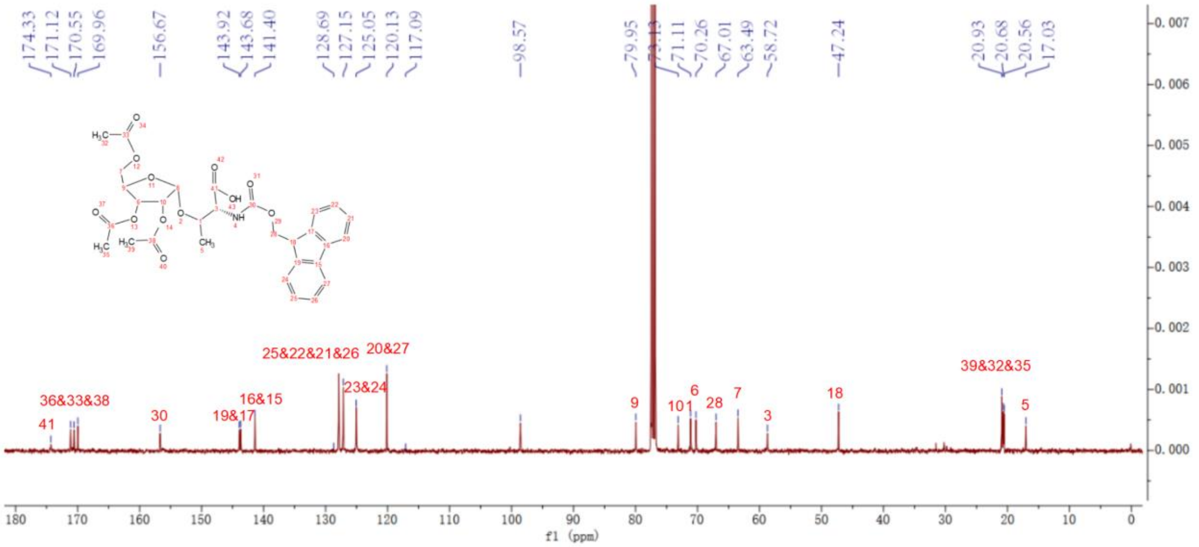

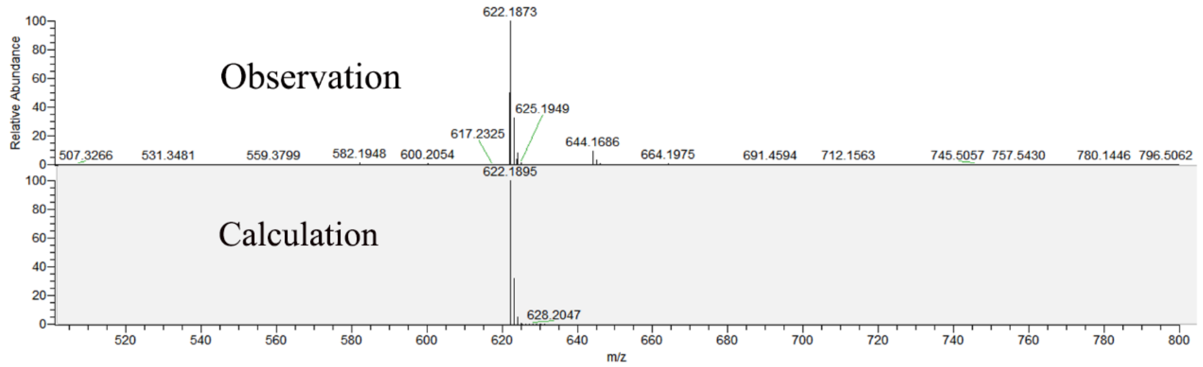

Figure S35. ^{1}H-NMR, ^{13}C-NMR, ESI-MS of **Fmoc-D-Thr-[β-D-Rib(Ac3)]-OH.** (^{1}H NMR (400 MHz, Chloroform-*d*)-- δ 7.76 (d, *J* = 7.6 Hz, 2H), 7.60 (t, J = 7.71 Hz, 2H), 7.39 (t, *J* = 7.5 Hz, 2H), 7.29 (m, 2H), 5.61 (d, *J* = 8.9 Hz, 1H), 5.33 (d, *J* = 4.4 Hz, 1H), 5.22 – 5.15 (m, 1H), 4.92 – 4.86 (m, 1H), 4.49 – 4.39 (m, 3H), 4.29 – 4.21 (m, 3H), 4.19 – 4.10 (m, 1H), 2.09 (s, 3H), 2.07 (s, 3H), 2.04 (s, 3H), 1.19 (d, *J* = 6.3 Hz, 3H); ^{13}C NMR (100 MHz, Chloroform-*d*)-- δ 174.33, 171.12, 170.55, 169.96, 156.67, 143.92, 143.68, 141.40, 128.69, 127.15, 125.05, 120.13, 117.09, 98.57, 79.95, 73.13, 71.11, 70.26, 67.01, 63.49, 58.72, 47.24, 20.93, 20.68, 20.56, 17.03). HRMS (ESI): m/z ([M+Na]+) calculated for $C_{30}H_{33}NO_{12}$: 622.1895; found: 622.1873.

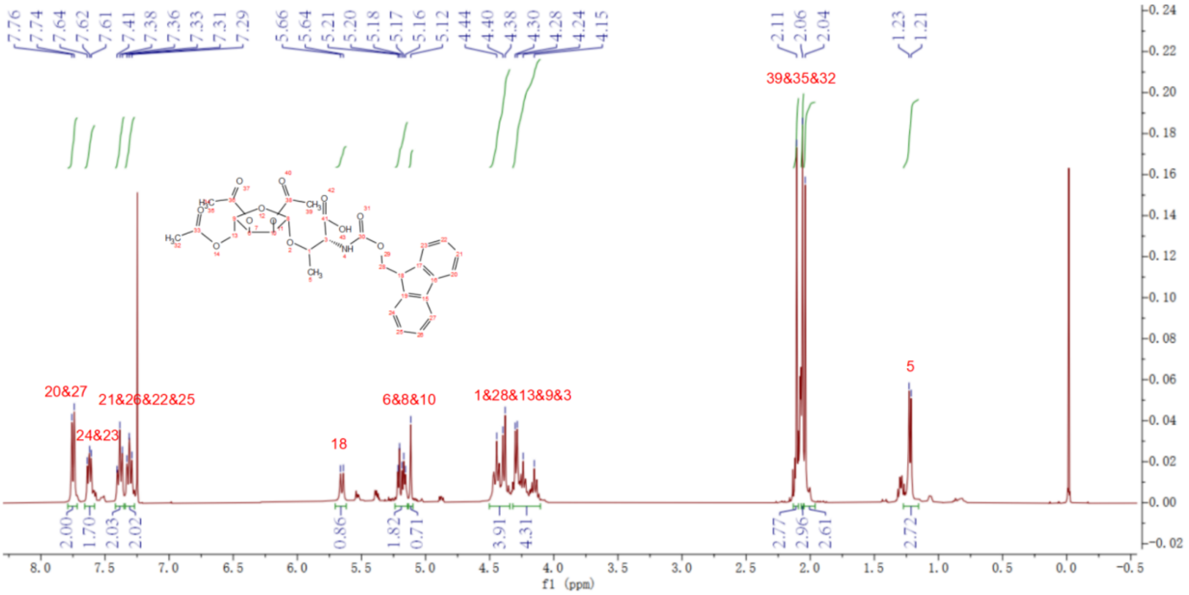

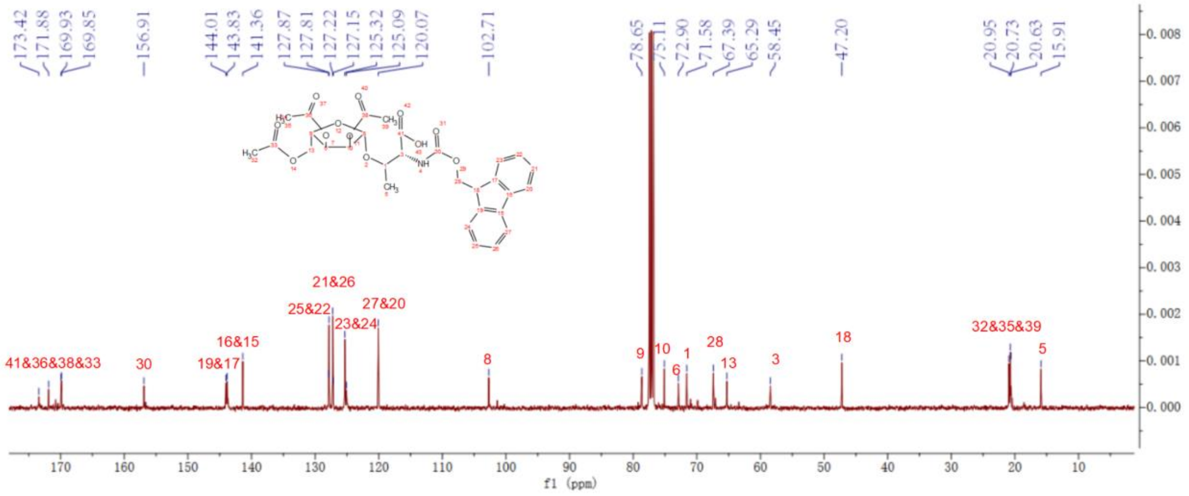

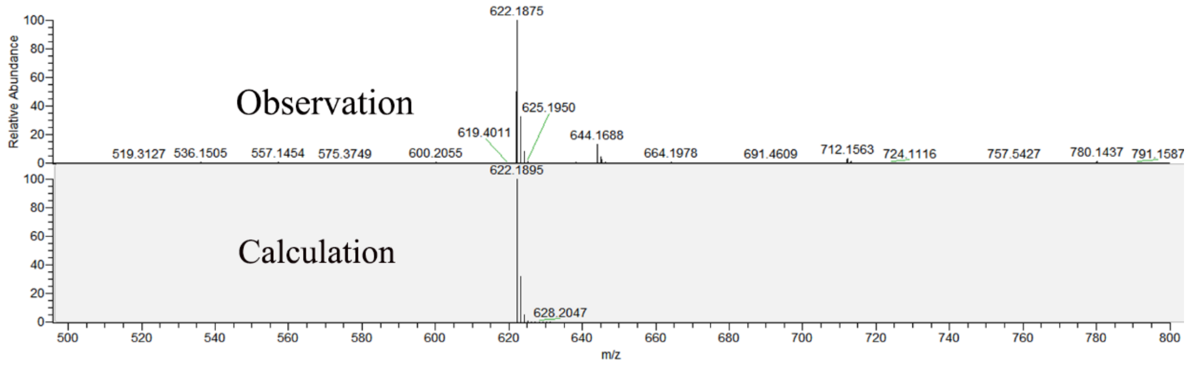

Figure S36. ^{1}H-NMR, ^{13}C-NMR, ESI-MS of **Fmoc-D-Thr-[β-L-Rib(Ac3)]-OH.** ^{1}H NMR (400 MHz, Chloroform-*d*)-- δ 7.75 (d, *J* = 7.4 Hz, 2H), 7.66 – 7.58 (m, 2H), 7.39 (t, *J* = 8.3 Hz, 2H), 7.31 (t, 2H), 5.65 (d, *J* = 9.2 Hz, 1H), 5.24 – 5.14 (m, 2H), 5.12 (s, 1H), 4.50 – 4.34 (m, 4H), 4.32 – 4.10 (m, 4H), 2.11 (s, 3H), 2.06 (s, 3H), 2.04 (s, 3H), 1.22 (d, *J* = 6.3 Hz, 3H); ^{13}C NMR (100 MHz, Chloroform-*d*)-- δ 173.42, 171.88, 169.93, 169.85, 156.91, 144.01, 143.83, 141.36, 127.87, 127.81, 127.22, 127.15, 125.32, 125.09, 120.07, 102.71, 78.65, 75.11, 72.90, 71.58, 67.39, 65.29, 58.45, 47.20, 20.95, 20.73, 20.63, 15.91. HRMS (ESI): m/z ([M+Na]$^+$) calculated for $C_{30}H_{33}NO_{12}$: 622.1895; found: 622.1875.
